# Impact of a *Saccharomyces cerevisiae* Fermentation Product Supplemented from 20 Days Before Dry-Off Through 60 Days of Lactation on the Metabolic Adaptation of Dairy Cows to the Peripartum Phase

**DOI:** 10.3390/ani15040480

**Published:** 2025-02-08

**Authors:** Matteo Mezzetti, Alessandro Maria Zontini, Andrea Minuti, Ilkyu Yoon, Erminio Trevisi

**Affiliations:** 1 Department of Animal Sciences, Food and Nutrition (DIANA), Facoltà di Scienze Agrarie, Alimentari e Ambientali, Università Cattolica del Sacro Cuore, 29122 Piacenza, PC, Italy; matteo.mezzetti@unicatt.it (M.M.); andrea.minuti@unicatt.it (A.M.); 2 Cargill Animal Nutrition and Health, 29017 Fiorenzuola d’Arda, PC, Italy; alessandro_zontini@cargill.com; 3 Diamond V, Cedar Rapids, IA 52404, USA; iyoon@diamondv.com

**Keywords:** inflammation, mammary gland, mastitis, postbiotics

## Abstract

Sixty Holstein cows were enrolled at −76 days from calving (DFC) and classified based on the daily SCC during the previous week from an automated milking system. The separation thresholds for low (L, *n* = 46) and high (H, *n =* 14) classifications were 100 K/mL for primiparous and 200 K/mL for multiparous cows. Cows were then assigned to two homogeneous groups to receive diets supplemented with 19 g/d of a *Saccharomyces cerevisiae* fermentation product (TRT; NutriTek, Diamond V, Cedar Rapids, IA, USA) or without supplementation (CTR) until 60 DFC. Cows were dried off at −56 DFC and monitored for disease incidence, milk yield and composition, plasma metabolic profile, and whole blood count from −76 to 60 DFC. Data were analyzed utilizing ANOVA and mixed models for repeated measures. During the dry period, TRT cows had greater plasma thiol and albumin compared to CTR. TRT-L cows had greater plasma protein and globulin than CTR-L. TRT-H cows had heightened hematocrit; reduced plasma globulin and haptoglobin; and higher albumin, albumin to globulin ratio, and thiol than CTR-H. TRT-H cows had greater concentrations of leukocytes and lymphocytes and lower plasma protein and ceruloplasmin at −54 DFC; lower reactive oxygen species to ferric ion-reducing antioxidant power ratios at −44 DFC; and greater concentrations of lymphocytes and plasma gamma glutamyl transferase at −7 DFC than CTR-H. After calving, TRT cows had a lower incidence of mastitis and higher butterfat, as well as greater plasma haptoglobin and aspartate amino transferase (AST) and reduced Mg compared to CTR. TRT cows had lower SCC between 1 and 7 DFC and a greater ECM between 41 and 60 DFC compared to CTR. TRT-H cows had lower SCC between 1 and 7 DFC and greater hemoglobin and plasma AST than CTR-H. Ameliorated immune system functions due to *Saccharomyces cerevisiae* fermentation product administration lowered the SCC in TRT-H cows and prevented the onset of new intramammary infections across both L and H SCC groups, supporting the improved productive performance of dairy cows.

## Simple Summary

The impaired health status of the mammary gland at dry-off boosts leukocyte activities occurring during the involution phase, potentially affecting the successful adaptation of cows to new lactation through inducing a chronic inflammatory condition. A substantial number of studies have demonstrated the effectiveness of a feed additive containing *Saccharomyces cerevisiae* fermentation products in ameliorating udder health and lowering somatic cell counts of dairy cows at various stages of lactation. Despite that, none of the research aimed at investigating the potential of *Saccharomyces cerevisiae* fermentation products to improve the adaptation of the cows to the new lactation has started feeding the product prior to dry-off. To fill our knowledge gap, the present research was aimed at testing whether supplementing *Saccharomyces cerevisiae* fermentation products starting before dry-off to dairy cows could improve their metabolic adaptations during the dry phase and subsequent lactation. In addition, this study aimed to evaluate whether the milk somatic cell count status at the end of lactation influences the effect of *Saccharomyces cerevisiae* fermentation product supplementation. Supplementing *Saccharomyces cerevisiae* fermentation products before dry-off mitigated the systemic inflammatory conditions of dairy cows with greater somatic cell counts at the end of lactation. Furthermore, it prevented new intramammary infections affecting the cows in the subsequent early lactation period irrespective of their somatic cell count status before dry-off.

## 1. Introduction

After dry-off, the contribution of activated leukocytes to mammary renewal processes creates systemic inflammatory conditions and altered redox balance like those experienced by dairy cows around parturition [1,2,3]. Impaired mammary health at dry-off boosts the severity of leukocyte activities occurring at the cessation of milking, predisposing dairy cows to develop chronic inflammatory conditions [4]. Chronic inflammation has the potential to boost the magnitude of immune dysfunctions around calving and further impair the function of leukocytes at the onset of the next lactation [5]. Dysfunctional leukocytes offer ineffective protection against new intramammary infections, while mammary gland epithelial barriers are challenged by the sharp increase in milk production, making the first 2 months of the new lactation the greatest risk period for clinical mastitis [6,7]. Even though there is no consensus on this, several authors retain somatic cell count (SCC) as the most reliable marker of the health status of the mammary gland to adopt in the field [8], and SCC thresholds are currently utilized to choose the best dry-off approach for late-lactating cows to minimize health issues (i.e., selective dry cow therapy vs. antibiotic treatment) [9].

*Saccharomyces cerevisiae* fermentation product (SCFP) is a proprietary mixture of bioactive compounds including yeast fermentation end-products, β-glucans, polyphenols (an antioxidant), and B-vitamins. Feeding SCFP to dairy cows has mitigated systemic inflammation and oxidative stress conditions [10] and improved leukocyte functions [11]. Several studies have suggested that these responses reduce the incidence of metabolic [12] and infectious diseases [13] in dairy cows receiving SCFP. Reduced mastitis incidence and improved udder health have been consistently demonstrated as major effects of SCFP [13,14], and a nutrigenomic mechanism has been proposed to account for the positive effects on the mammary glands [15].

Despite a substantial number of studies evaluating the effects of SCFP, none of the research has started feeding the product prior to dry-off. To fill our knowledge gap, the present research was aimed at testing whether supplementing SCFP starting before dry-off to dairy cows could improve their metabolic adaptations during the dry phase and subsequent lactation. Given the documented potential of SCFP to support immune function and udder health, this study aimed to determine whether SCC status at the end of lactation influences the response to SCFP supplementation, thereby providing insights into its targeted application in dairy herds. Our hypothesis was that SCFP supplementation before dry-off could improve the metabolic adaptation of dairy cows to the peripartum phase, and that SCC level before dry-off could impact the metabolic response to the dietary treatment.

## 2. Materials and Methods

### 2.1. Animal Management

The study was conducted on a commercial dairy farm (Cascina Motta, Viadana di Calvisano, Brescia, Italy) from 4 April 2022 to 21 February 2023. The farm consisted of 210 milking cows that averaged 38.8 kg/d of milk (4.3% butterfat, 3.45% protein and 233 K/mL SCC at the bulk tank). Cows were housed in a freestall barn with headlocks and stocking density of 0.87 headlocks/cow and 0.97 stalls/cow. Cows were milked using 4 automated milking system stations (AMS; Lely Astronaut A4, Lely Industries N.V., Maassluis, The Netherlands) located in the middle of the lactation pen and allowing a free flow to the cows. Milking settings adopted by AMS are detailed in Table S1. All AMS stations were installed between 2011 and 2012; thus, cows were completely trained for the AMS system when the experiment was performed. At 64 days prior to the expected calving day, access to the AMS station was limited to 1 visit/d for cows yielding more than 20 kg milk/d to support an easier dry-off. At 60 days before expected calving day, cows were dried off after being thoroughly milked with a bucket unit and infused with an intramammary antibiotic (Mamyzin A; Haupt Pharma Latina S.r.l, Borgo San Michele-Latina, Italy), then moved to a straw-bedded pen. At 15 days before the expected calving day, cows were moved to a close-up straw-bedded pen to allow for a comfortable environment for parturition. After parturition, cows were moved to a postpartum straw-bedded pen until 20 days from calving (DFC), at which time they were automatically shifted to the lactation group by the AMS station. Stocking density was at least 1.05 headlocks/cow when cows were housed in straw-bedded pens of the dry, close-up, and postpartum groups.

### 2.2. Experimental Design

A group of 60 parous Italian Holstein dairy cows (parity: 1.93 ± 1.0; BW: 757 ± 89 kg; BCS: 2.96 ± 0.39; average lactation length: 285 ± 31 days [mean ± SD]) was enrolled in the experiment at −76 ± 2.0 DFC and dried off at −56 ± 4 DFC. No pregnant heifers approaching their first calving were enrolled in this experiment. On the enrollment day, experimental cows were classified as high (H) or low (L) based on the average of the daily SCC values reported by the AMS computer system from −83 to −76 DFC (SCC_LV). The separation thresholds for low (L, *n =* 46) and high (H, *n =* 14) classifications were 100 K/mL for primiparous and 200 K/mL for multiparous cows. Thresholds were defined based on those proposed by Vanhoudt et al. (2018) [9] (i.e., 100 K/mL for primiparous and 250 K/mL for multiparous cows) but reduced to meet the current recommendations adopted in Italian herds performing a selective dry-off procedure. Cows were randomly assigned to two experimental dietary groups blocked by SCC_LV; parity; calving interval; estimated milk yield for a 305-d lactation and BCS at −76 DFC; and daily averages of milk yield, milk composition, body weight, and minutes of rumination measured between −83 and −76 DFC (Table 1). The randomization process consisted of pairing the cows eligible for enrollment based on their scheduled dry-off day. At the enrollment, each cow within a couple was allocated to CTR or TRT groups, minimizing the differences existing among the two experimental groups at the baseline for the parameters included in Table 1. The same procedure was adopted for all the cows included in the experiment until the final sample size was reached. Cows in the two experimental dietary groups received diets either supplemented with 19 g/d of a *Saccharomyces cerevisiae* fermentation product (TRT; NutriTek^®^, Diamond V; *n =* 30 cows of 7 H; 23 L) or without supplementation (CTR; *n =* 30 cows of 7 H; 23 L) from −76 to 60 DFC. The exact components and composition of the SCFP used in this experiment are proprietary and not publicly available.

### 2.3. Experimental Diets Formulation and Administration

During the experiment, forages were analyzed by near-infrared spectroscopy at each change of the batches, and diets were formulated using the MAX™ System for Dairy (Cargill, Inc., Fiorenzuola d’Arda, Italy) to meet the nutrient requirements of the cows (Table 2). During the dry and close-up phases, cows in the CTR and TRT groups were housed in separate pens and fed a total mixed ration (TMR). The SCFP was hand-added to the mixer wagon and mixed into the TMR diets of TRT cows. Daily TMR distribution was adjusted to ensure 5% leftovers were always maintained. The amount of SCFP included in the TMR was adjusted based on the daily distribution to allow for a constant consumption of 19 g SCFP/cow/d. TMR diets were always prepared for a minimum of 50 cows to accommodate the minimum capacity of the mixer wagon to ensure accurate mixing. During the lactation phase, cows in the CTR and TRT groups were housed together, receiving a partial mixed ration (PMR) and up to 6 kg/d (average of 5 kg/d, as-fed basis) of a milking robot concentrate supplemented by the AMS station (Table S1). SCFP was delivered through the AMS stations according to Zontini et al. (2021) [14]. Briefly, 2 nutritionally identical concentrates were formulated for a 650 kg cow producing 38 kg/d of milk at 4.0% fat and 3.4% protein and eating 24.13 kg/d of DM, then delivered by the AMS stations through separate lines. CTR cows received an appropriate amount of concentrate without SCFP adjusted to their milk production. TRT cows received 2.0 kg/d (evenly distributed throughout the day) of a concentrate containing 19 g/d of SCFP, and their remaining daily requirements were met with an appropriate amount of concentrate from the CTR line. Refusals were visually checked for each cow independently to make sure the SCFP was consumed completely at the AMS station.

### 2.4. Animal Measurements, Samples Collection, and Handling Procedures

**Figure 1:**
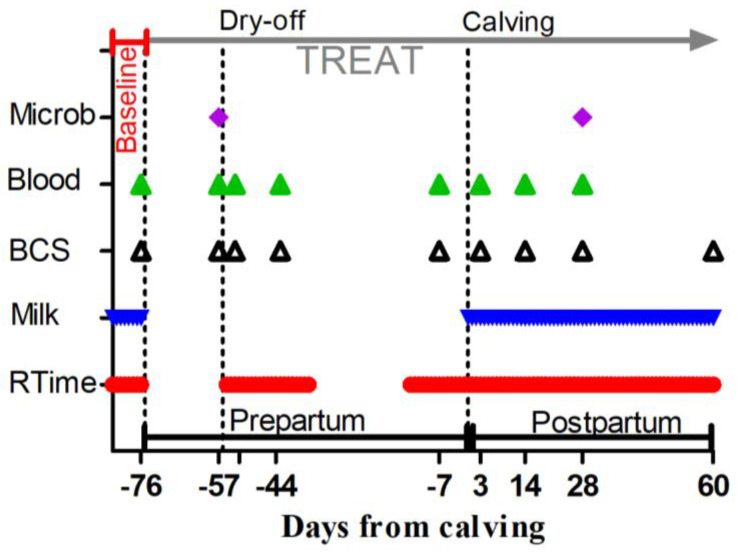
Scheduled time points, expressed as days from calving, for the administration of a *Saccharomyces cerevisiae* fermentation product or a control diet (TREAT), rumination time measurement (RTime), milk yield, butterfat, protein, lactose and SCC recordings (Milk), body condition scoring (BCS), blood sample collection for whole-blood glutathione peroxidase, plasma metabolic profile, complete blood cell count determination (Blood), and milk sample collection for mammary gland bacteriology assessment (Microb). Baseline indicates the samples used to determine the baseline for assessed parameters. Dry-off is day when milk removal was halted. Calving is day of delivery.

Periodical sampling and data collections were performed on cows from −83 to 60 DFC, as shown in Figure 1 and described in the following sections.

#### 2.4.1. Health Status

During the study, the health statuses of the cows were monitored daily, and all veterinary interventions that occurred from –83 to 60 DFC were recorded. Metritis, endometritis, and pyometra were diagnosed by a veterinary partitioner according to prior guidelines [16]. Endometritis and metritis were treated with tetracycline injection (Oxtra; Fatro SP, Ozzano dell’Emilia, Italy). Pyometra was treated with a uterine flush and 2 injections of prostaglandins (Estrumate^®^; MSD Animal Health, Rahway, NJ, USA) combined with tetracycline (Panterramicine^®^; Zooetis, Rome, Italy or Oxtra; Fatro SP), sulfonamide (Daipirim; Izo SRL, Cuxhaven, Germany), or penicillin therapy (Repen; Fatro SP). Other diseases were diagnosed by the farmer. Milk fever was diagnosed when the cow was unable to stand on her feet after delivery and treated with intravenous calcium solution until recovery. Retained placenta was diagnosed when the fetal membranes were not expelled within 12 h after calving and treated with oxytocin (Neurofisin; Fatro SP) and penicillin (Procactive; Laboratoryos Syva S.A., León, Spain) combined with tetracycline as needed (Oxtra; Fatro SP). Ketosis was diagnosed based on the BHB detected in urine at 3 DFC with a commercial kit (PV290798; Profilab urine, Professional Vet, Lodi, Italy), and a threshold of 1.5 mmol/L was used to indicate treatment with a glucose solution until recovery. Mastitis was diagnosed by the AMS computer system when milk SCC remained above the 200 K/mL threshold for 24 h, and diagnosis was visually confirmed by the farmer. Mastitis was treated with systemic anti-inflammatory (Fatrocortin; Fatro SP or Novasterol; Ceva SPA, Lazio, Italy) or anti-pyretic (Farmolisine; Ceva SPA) injections combined with intramammary treatment with tetracycline (Oxtra; Fatro SP), penicillin (Procactive; Laboratoryos Syva S.A.), or cephalosporin (Cefaximin; Fatro SP) until complete recovery, as indicated by the California mastitis test. Treatment of uterine diseases and mastitis was always preceded by a microbial screening to identify the most effective antimicrobial protocol against the bacteria based on antibiograms performed monthly within the herd.

#### 2.4.2. Rumination Time, Milk Yield, and Composition and Somatic Cell Count

Between –83 and 60 DFC, the rumination time, milk yield, milk composition (butterfat, protein, and lactose concentration), and SCC of the cows were recorded daily by each AMS station through the MQC-C system (Lely Qwes-HR collars, Lely Industries N.V., Maassluis, The Netherlands). The algorithms of the AMS stations for butterfat, protein, lactose, and SCC were calibrated relative to the analytical values of milk samples collected from the bulk tank once a week and from individual cows once a month.

#### 2.4.3. Milk Samples

At −57 DFC (the day before dry-off), a sterile milk sample was collected from cows in the morning following standard procedures provided by the National Mastitis Council (1999) [17] with some modifications. Briefly, while cows were restrained at the feeding bunk headlocks, the mammary gland was stimulated, and the first streams of milk were discarded. Subsequently, the teats were dipped in iodine tincture, cleaned, and disinfected using 70% ethanol. Samples were then collected by pooling milk from each mammary quarter into sterile plastic tubes without preservatives (Corning Life Sciences, Tewksbury, MA, USA) and immediately sent to “Istituto Zooprofilattico Sperimentale della Lombardia e dell’Emilia-Romagna Bruno Umbertini” (Strada della Faggiola, 1, 29027, Gariga, PC, Italy) for assessment of the mammary gland bacteriology.

#### 2.4.4. Body Condition Score and Blood Samples

At −76, −57, −54, −44, −7, 3, 14, 28, and 60 ± 1 DFC, the cow’s BCS was determined by the same operator using a 1 to 4 scale [18]. At the same time points (excluding 60 DFC), two blood samples were collected from cows before the morning feeding by means of jugular venipuncture with K-EDTA and heparinized evacuated collection tubes (BD Vacutainer, Becton, Dickinson and Company, Franklin Lakes, NJ, USA). K-EDTA blood samples were immediately sent to Istituto Zooprofilattico Sperimentale della Lombardia e dell’Emilia-Romagna “Bruno Umbertini” (Strada della Faggiola, 1, 29027, Gariga, PC, Italy) for assessing the complete blood cell count. A 20 µL aliquot of heparinized whole blood was used to assess glutathione peroxidase (GPx) according to recommendations provided by a commercial kit (Ransel kit, Randox, Crumlin, UK), with some modifications. Briefly, whole blood was aseptically removed, immediately diluted with the provided reagent, and frozen at −20 °C. Remaining heparinized blood was processed according to Calamari et al. (2016) [19]. Blood was centrifuged, and plasma was frozen at −20 °C to assess the metabolic profile.

### 2.5. Analytical Procedures

Frozen samples used to perform the analytical procedures were thawed and processed as described below. The average storage time was 3 months for whole blood and plasma samples. Other samples were analyzed within 24 h of collection.

#### 2.5.1. Mammary Gland Bacteriology

*Staphylococcus* spp., *Bacillus* spp., *Streptococcus dysgalactiae*, *Streptococcus uberis,* and mixed bacterial growth were determined by real-time PCR. *Escherichia coli* and yeasts were determined by incubation of milk samples on a selective culture medium according to Adkins et al. (2017) [20]. Briefly, 10 μL of milk was spread onto plates of blood agar, Thallium Kristal Violette Toxin, and Gassner Agars (selective media for Streptococci and Enterobacteriaceae, respectively). Plates were incubated at 37 °C for 72 h and examined daily.

#### 2.5.2. Plasma Metabolic Profile

A clinical autoanalyzer (ILAB Taurus, Instrumentation Laboratory, Bedford, MA, USA) was used to determine the plasma concentrations of glucose, NEFA, BHB, urea, creatinine, calcium, phosphorus, magnesium, zinc, aspartate amino transferase–glutamate oxaloacetate transaminase (AST), gamma glutamyl transferase (GGT), alkaline phosphatase, total protein, haptoglobin, ceruloplasmin, albumin, total bilirubin, and cholesterol in accordance with Calamari et al. (2016) [19]. The plasma concentrations of paraoxonase, reactive oxygen metabolites (ROMt), and ferric ion-reducing antioxidant power (FRAP) were determined according to Mezzetti et al. (2019) [21], those of thiol according to Minuti et al. (2014) [22], those of myeloperoxidase according to Bradley et al. (1982) [23], and the whole-blood GPx concentration according to Calamari et al. (2007) [24]. A multidetector microplate reader (Synergy 2, BioTek Instruments, Winooski, VT, USA) and commercial kits for the ELISA methods were used to determine the plasma concentrations of IL-6 (cat. no. ESS0029; Thermo Fisher Scientific, Frederick, MD, USA), according to Mezzetti et al. (2019) [21], and insulin (cat. no. 10-1201-01; Mercodia AB; Uppsala, Sweden). The plasma concentrations of retinol, tocopherol, and beta-carotene were analyzed by reverse-phase high-performance liquid chromatography (LC-4000, Jasco Europe SRL, Cremella, Italy), as described by Minuti et al. (2014) [22]. Further details on the analytical procedures of the metabolic profile analysis are reported in Table S2.

#### 2.5.3. Complete Blood Cell Count

Samples were processed with a clinical autoanalyzer (Cell-DYN 3700, Abbott Diagnostics, Santa Clara, CA, USA). A laser optics assay was used to determine the total counts of white blood cells (WBC), monocytes, basophils, eosinophils, neutrophils, and lymphocytes. The mean cell volume (MCV), hematocrit, number of red blood cells, mean red blood cell distribution wide (RDW) and number of platelets were determined via an electrical impedance assay. The hemoglobin, mean cell hemoglobin (MCH), and mean cell hemoglobin concentration (MCHC) were determined using a spectrophotometry assay.

#### 2.5.4. Calculations

In milk, the SCC was expressed as a somatic cells score (SCS) according to Wiggans and Shook (1987) [25], and the energy-corrected milk (ECM) was calculated based on daily yield and butterfat, protein, and lactose contents according to Sjaunja et al. (1990) [26]. Plasma globulin content was calculated as the difference between total protein and albumin. Among plasma analyte ratios, the NEFA to albumin ratio (NAR) was calculated according to Gonçalves-de-Albuquerque et al. (2019) [27]; the albumin to globulin ratio (AGR) according to Cattaneo et al. (2021) [3]; the ROMt to FRAP ratio (RFR) according to Ling et al. (2018) [28]; and the myeloperoxidase to paraoxonase ratio (MPR) according to Haraguchi et al. (2014) [29]. Among plasma indexes, the quantitative insulin sensitivity check index (QUICKI), the revised QUICKI (RQUICKI), and the RQUICKI accounting for BHB contribution (RQUICKIBHB) were calculated based on the concentrations of insulin, glucose, NEFA, and BHB according to Katz et al. (2000) [30] and De Koster and Opsomer (2013) [31]. The liver functionality index (LFI) was calculated based on the concentrations of albumin, cholesterol, and bilirubin according to Zontini et al. (2012) [14].

### 2.6. Statistical Analysis

The number of animals enrolled in this study (*n =* 30 cows/group) was calculated on SCC at enrollment to ensure a statistical power > 0.8 for detecting differences of 15 K cells/mL (i.e., SCS = 0.26) among groups, considering α = 0.05 and a standard deviation of 461 K cells/mL (i.e., SCS = 1.38) (Power Procedure, SAS Inst. Inc.). As SCC classification reflected the actual prevalence of low (L) and high (H) SCC animals within the commercial farm, a power analysis was conducted for the H SCC cows (*n =* 7 cows/sub-group) to minimize any potential bias in the results caused by the small sample size in this sub-classification. The analysis considered a standard deviation of 527.8 K cells/mL (i.e., SCS = 1.54), with α = 0.05, resulting in a power of 0.64.

The data were analyzed using SAS software, version 9.4 (SAS Inst. Inc., Cary, NC, USA), and are presented in graphs and tables as the least squares mean and pooled standard error for individual means of treatment over time. The prevalence of health diagnoses was evaluated by *χ2* analysis (Freq procedure, SAS Institute, Inc., Cary, NC, USA).

Data on the rumination time, milk yield, milk composition, SCC, BCS, and blood analytes underwent ANOVA testing using a mixed model for repeated measures (Glimmix Procedure, SAS Inst. Inc.). For rumination time, milk yield, milk composition, SCC, BCS, and blood analytes, the statistical model included the fixed effects of treatment (TREAT; CTR and TRT groups), SCC_LV (L and H), and time; the first-order interaction effects of TREAT × SCC_LV and TREAT × time; the second-order interaction effect of TREAT × SCC_LV × time; and the random effect of the individual cow.

*yijkl* = *µ* + *αi* + *δij* + *βk* + *εkj* + *τl* + *(αβ)ik* + *(ατ)il* + *(αβτ)ikl* + *eijkl*

where *yijkl* is the response at time *l* on animal *j* in TREAT *i* with the SCC_LV *k*; *µ* is the overall mean; *αi* is a fixed effect of TREAT *i; δij* is a random effect of animal *j* in TREAT *i; βk* is a fixed effect of SCC_LV *k*; *εkj* is a random effect of animal *j* in SCC_LV *k*; *τl* is a fixed effect of time *l*; (*αβ*)*ik* is a fixed first-order interaction effect of TREAT *i* with SCC_LV *k*; (*ατ*)*il* is a fixed first-order interaction effect of TREAT *i* with time *l*; (*αβτ*)*ikl* is a fixed second-order interaction effect of TREAT *i*, time *l*, and SCC_LV *k*; and *eijkl* is random error at time *l* on animal *j* in TREAT *i* with the SCC_LV *k*.

Time effect had 8 observations for BCS (−57, −54, −44, −7, 3, 14, 28, and 60 DFC) and 7 observations for blood analytes (−57, −54, −44, −7, 3, 14, and 28 DFC). For rumination time, time effect considered daily values grouped into 7 experimental phases (Ph1 = from −55 to −35 DFC; Ph2 = from −14 to 0 DFC; Ph3 = from 1 to 7 DFC; Ph4 = from 8 to 21 DFC; Ph5 = from 22 to 40 DFC; Ph6 = from 41 to 60 DFC). For milk yield, milk composition, and SCC, the time effect considered 4 experimental phases only, as Ph1 and Ph2 were excluded. The BY statement was added to the model to split the statistical analysis into two independent periods relative to parturition (prepartum and postpartum). The analyses were conducted using four covariance structures—autoregressive, unstructured, antedependence, and spatial power—with their heterogeneous counterparts. These were ranked according to their Akaike information criteria, with the one having the lowest criterion being chosen for final statistical analysis [32]. Data on LFI underwent ANOVA testing using a mixed model including the fixed effect of TREAT and SCC_LV and the first-order interaction effect of TREAT × SCC_LV only.

A preliminary analysis was conducted, and all parameters were covariate using data collected before treatment administration as the baseline (i.e., between −83 and −76 DFC for rumination time, milk yield, milk composition and SCC and at −76 DFC for BCS and blood analytes), assuming *p* < 0.1 as a cutoff for covariate inclusion in the model. None of the parameters considered required covariate inclusion, and thus, baseline values were dropped from the final model.

The distribution of residuals was visually assessed. The pairwise comparison was performed using the least significant difference test with the Tukey adjustment for multiple comparisons. Significance was declared for *p* ≤ 0.05, and differences for *p* ≤ 0.1 were discussed in the context of tendencies.

## 3. Results

Parameters affected by TREAT, SCC_LV, or the two-way interaction of TREAT × SCC_LV will be presented in tables as LS-MEANS and SEM. Tables will include overall LS-MEANS and SEM for each period separately for parameters with multiple measurements in the prepartum and postpartum periods. Time effects will not be presented in the results section or discussed thereafter. Parameters affected by the two-way interaction TREAT × Time or by the full interaction TREAT × SCC_LV × Time will be presented as figures.

### 3.1. Mammary Gland Bacteriology and Health Status

None of the cows included in the study were affected by any contagious pathogenic bacteria before dry-off (Table 3).

**Figure 2:**
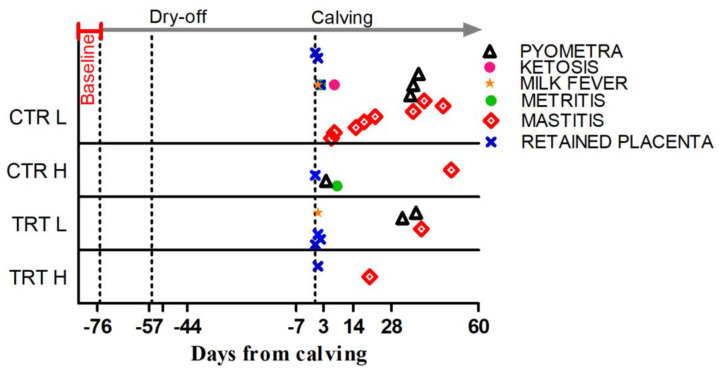
Occurrence, expressed as days from calving, for the metabolic and infectious diseases registered during the experiment in the 4 experimental groups. Baseline indicates the pre-enrollment phase. Dry-off is day when milk removal was halted. Calving is day of delivery.

The DFCs at which diseases were diagnosed are shown in Figure 2.

Regardless of SCC_LV classification and among L cows, TRT group had a greater incidence of healthy cows and a reduced incidence of mastitis compared to CTR (Table 4). No effect was detected for the incidence of other diseases for the dietary supplement or SCC groups.

### 3.2. Milk Yield and Composition, Body Condition Score, and Rumination Time

**Figure 3:**
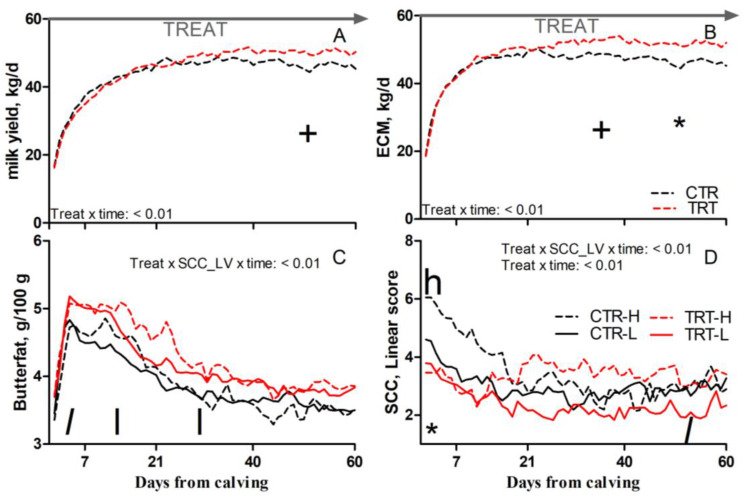
Pattern of milk yield (**A**), energy-corrected milk (ECM, (**B**)), milk butterfat (**C**), and milk somatic cell count (SCC; (**D**)) in dairy cows with low (L) or high (H) SCC between −27 and −20 days from dry-off and receiving 19 g/d of *Saccharomyces cerevisiae* fermentation product (TRT) or a control diet (CTR) from −20 days from dry-off to 60 days from calving (CTR-L = 23 cows, CTR-H = 7 cows, TRT-L = 23 cows, TRT-H = 7 cows). “TREAT” indicates timing for experimental diet administration. Comparisons at each time point are indicated with different symbols for the treatment × time interaction (Treat × time; * is *p* < 0.05; + is *p* < 0.1) and letters for the treatment × SCC level × time interaction (Treat × SCC_LV × time; “*l*” is *p* < 0.1 and “l” is *p* < 0.05 for L cows; “h” is *p* < 0.05 for H cows).

Compared to L and regardless of dietary supplementation, H cows had a tendency toward greater SCC (*p* = 0.1; Table 5). Compared to CTR and regardless of SCC_LV classification, the TRT group had greater butterfat (*p* = 0.03; Table 5); a tendency toward greater milk yield between 41 and 60 DFC (*p* < 0.1; Figure 3A); a tendency toward greater ECM between 22 and 40 DFC and greater ECM between 41 and 60 DFC (*p* < 0.1 and 0.05, respectively; Figure 3B); and reduced SCC between 1 and 7 DFC (*p* < 0.05; Figure 3D). Compared to CTR-L, TRT-L cows had a tendency toward greater butterfat between 1 and 7 DFC; greater butterfat between 8 and 40 DFC (*p* < 0.1 and 0.05, respectively; Figure 3C); and a tendency toward reduced SCC between 41 and 60 DFC (*p* < 0.1; Figure 3D). Compared to CTR-H, TRT-H cows had a reduced SCC between 1 and 7 DFC (*p* < 0.05; Figure 3D). No effects were detected for BCS, rumination time, milk protein, or lactose content due to dietary supplementation or SCC_LV (Table 5).

### 3.3. Plasma Metabolic Profile

#### 3.3.1. Energy, Protein, and Mineral Metabolism Biomarkers

**Figure 4:**
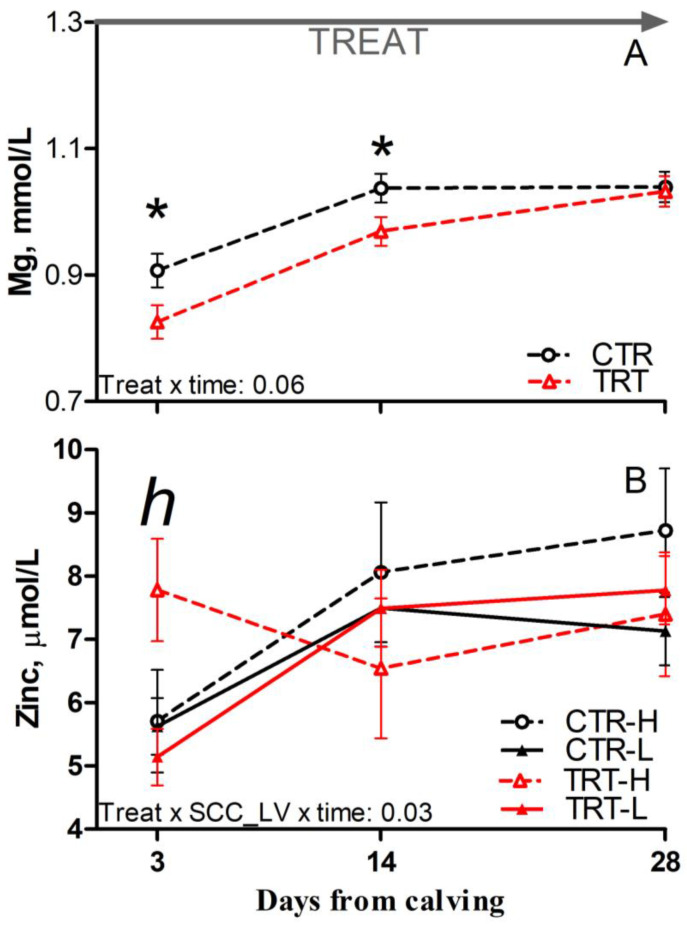
Postpartum time course of plasma concentrations of magnesium (**A**) and zinc (**B**) in dairy cows with low (L) or high (H) SCC between −27 and −20 days from dry-off and receiving 19 g/d of *Saccharomyces cerevisiae* fermentation product (TRT) or a control diet (CTR) from −19 days from dry-off to 60 days from calving (CTR-L = 23 cows, CTR-H = 7 cows, TRT-L = 23 cows, TRT-H = 7 cows). “TREAT” indicates timing for experimental diet administration. Comparisons at each time point are indicated with different symbols for the treatment × time (Treat × time; * is *p* < 0.05) and with different letters for the treatment × SCC level × time interaction (Treat × SCC_LV × time; “*h*” is *p* < 0.1 for H cows).

Compared to L and regardless of dietary supplementation, H cows had reduced glucose and a tendency toward reduced Mg concentration prepartum (*p* = 0.03 and 0.08, respectively; Table 6). Compared to CTR and regardless of SCC_LV classification, the TRT group had reduced Mg at 3 and 14 DFC (*p* < 0.05; Figure 4A). Compared to CTR-H, TRT-H cows had reduced urea postpartum (*p* < 0.05; Table 6) and a tendency toward reduced Zn at 3 DFC (*p* < 0.1; Figure 4B). No effect was detected for other analytes or indexes reflecting energy, protein, or mineral metabolism due to dietary supplementation or SCC_LV classification (Table S3).

#### 3.3.2. Inflammation and Liver Function Biomarkers

**Figure 5:**
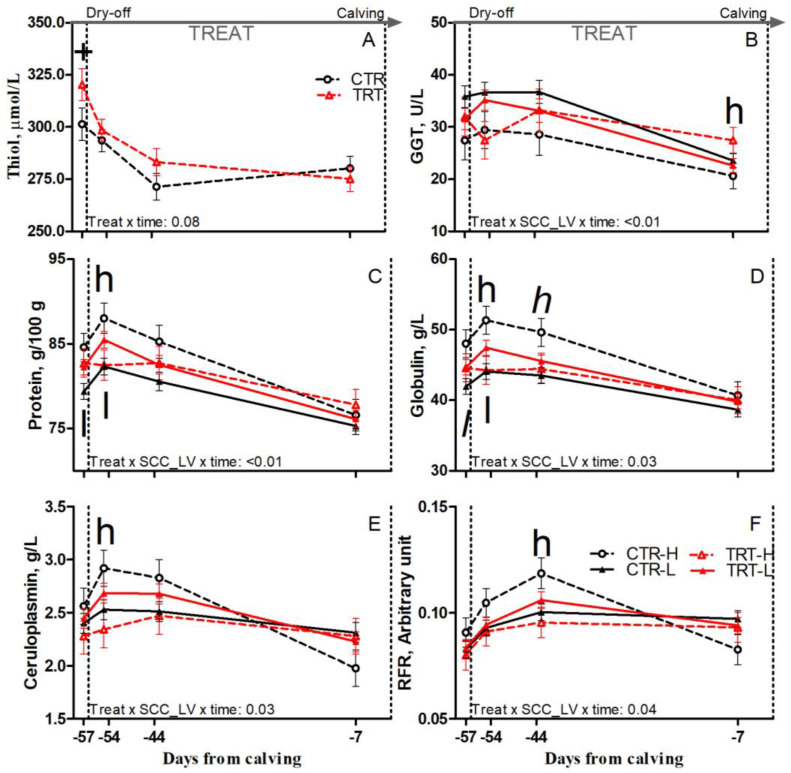
Prepartum time course of plasma concentrations of thiol (**A**), γ-glutamyl transferase (GGT; (**B**)), protein (**C**), globulin (**D**), ceruloplasmin (**E**), and reactive oxygen metabolites to ferric ion-reducing antioxidant power ratio (RFR; (**F**)) in dairy cows having a low (L) or high (H) SCC between −27 and −20 days from dry-off and receiving 19 g/d of *Saccharomyces cerevisiae* fermentation product (TRT) or a control diet (CTR) from −20 days from dry-off to 60 days from calving (CTR-L = 23 cows, CTR-H = 7 cows, TRT-L = 23 cows, TRT-H = 7 cows). “TREAT” indicates timing for experimental diet administration. Comparisons at each time point are indicated with different symbols for the treatment × time (Treat × time; + is *p* < 0.1) and with different letters for the treatment × SCC level × time interaction (Treat × SCC_LV × time; “*l*” is *p* < 0.1 and “l” is *p* < 0.05 for L cows; “*h*” is *p* < 0.1, and “h” is *p* < 0.05 for H cows).

Compared to L and regardless of dietary supplementation, H cows had tendencies toward greater protein and reduced cholesterol prepartum (*p* = 0.1 and 0.07, respectively), greater protein, and greater globulin concentrations postpartum (*p* < 0.01 and 0.06, respectively; Table 6). Compared to CTR and regardless of SCC_LV classification, the TRT group had greater albumin prepartum (*p* = 0.05) and greater AST and haptoglobin postpartum (*p* < 0.01 and 0.05, respectively; Table 6). Compared to CTR-L, TRT-L cows had tendencies toward reduced AGR postpartum (*p* < 0.1; Table 6), greater protein, and greater globulin at −57 DFC (*p* < 0.05 and 0.1, respectively), and had greater protein and globulin at −54 DFC (*p* < 0.05; Figure 5C,D). Compared to CTR-H, TRT-H cows had reduced haptoglobin and greater albumin and AGR prepartum (*p* < 0.05; Table 6); reduced protein, globulin, and ceruloplasmin at −54 DFC (*p* < 0.05; Figure 5C–E); and a tendency toward reduced globulin at −44 DFC (*p* < 0.1; Figure 5D). Nonetheless, the TRT-H group had greater GGT at −7 DFC (*p* < 0.05; Figure 5B) and greater AST postpartum than the CTR-H group (*p* < 0.01; Table 6). No effect was detected for other inflammation and acute phase biomarkers (Table S3) due to dietary supplementation or SCC_LV classification.

#### 3.3.3. Redox Balance Biomarkers

Compared to L and regardless of dietary supplementation, H cows had a tendency toward greater thiol concentration postpartum (*p* = 0.1; Table 6). Compared to CTR and regardless of SCC_LV classification, the TRT group had greater thiol prepartum (*p* = 0.04; Table 6), particularly at −57 DFC (*p* < 0.1; Figure 5A). Compared to CTR-H, TRT-H cows had greater thiol prepartum (*p* < 0.05; Table 6) and reduced RFR at −44 DFC (*p* < 0.05; Figure 5F). No effect was detected for the other oxidant species or body antioxidant indicators (Table S3) due to diet or SCC_LV classification.

### 3.4. Complete Blood Cell Count

**Figure 6:**
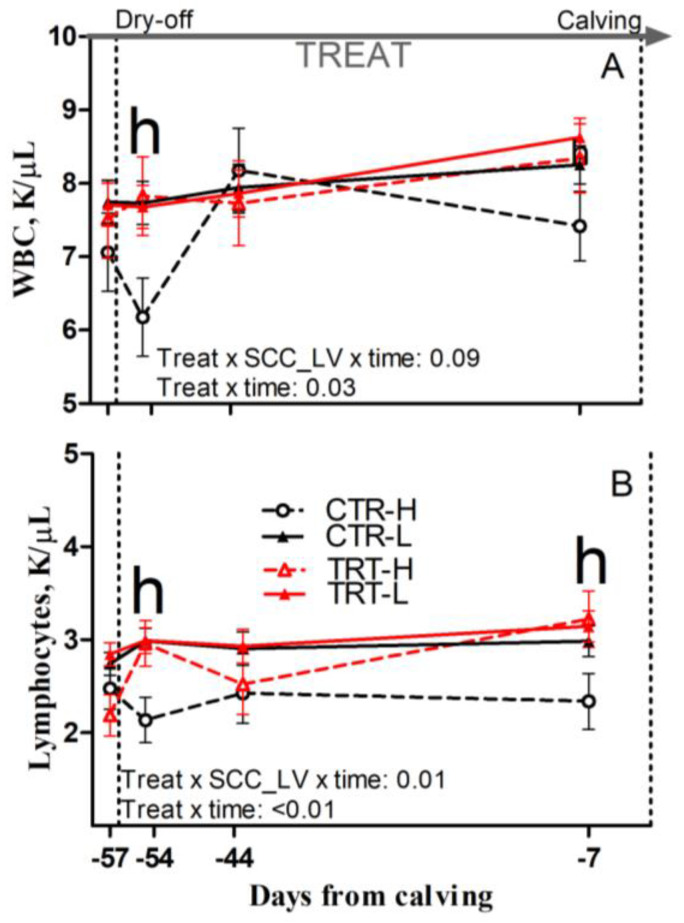
Prepartum time course of total white blood cells (WBCs; (**A**)) and blood lymphocytes (**B**) in dairy cows having low (L) or high (H) SCC between −27 and −20 days from dry-off and receiving 19 g/d of *Saccharomyces cerevisiae* fermentation product (TRT) or a control diet (CTR) from −19 days from dry-off to 60 days from calving (CTR-L = 23 cows, CTR-H = 7 cows, TRT-L = 23 cows, TRT-H = 7 cows). “TREAT” indicates timing for experimental diet administration. Comparisons at each time point are indicated with different letters for the treatment × SCC level × time interaction (Treat × SCC_LV × time; “h” is *p* < 0.05 for H cows).

Compared to L and regardless of dietary supplementation, H cows had a tendency toward greater RDW and had greater relative abundance of neutrophils, reduced lymphocyte concentrations and relative abundance prepartum (*p* = 0.08; 0.02; 0.04 and 0.02, respectively), and reduced monocyte concentrations postpartum (*p* = 0.03; Table 7). Compared to CTR and regardless of SCC_LV classification, the TRT group had greater RBC, hemoglobin, and hematocrit prepartum (*p* = 0.01, 0.01 and 0.02, respectively) and had a tendency toward greater hemoglobin postpartum (*p* = 0.06; Table 7). Compared to CTR-L, TRT-L cows had reduced monocytes and a tendency toward reduced basophil concentrations, as well as lower relative abundance of basophils postpartum (*p* < 0.05, 0.01 and 0.05, respectively; Table 7). Compared to CTR-H, TRT-H cows had greater hematocrit prepartum (*p* < 0.05; Table 7); greater WBC and lymphocyte concentrations at −54 DFC (*p* < 0.05; Figure 6A,B); greater lymphocyte concentrations at −7 DFC (*p* < 0.05; Figure 6B); and greater hemoglobin postpartum (*p* < 0.05; Table 7). No effect was detected for the other complete blood cell count parameters (Table S4) due to diet or SCC_LV classification.

## 4. Discussion

In this study, SCFP supplementation reduced the incidence of mastitis and milk SCC in cows following parturition, further confirming the effectiveness of SCFP in improving mammary gland health, which has been reported previously [13,14]. Importantly, the lower number of cows included in the H SCC group should be considered as a limitation of this study (as reflected by the power analysis conducted on SCC). This is especially true considering binary variables as the incidence of diseases, and the effect of SCFP in lowering the incidence of mastitis in L SCC cows only could be driven by this confounding effect. Greater milk SCC and clinical mastitis have been widely documented as predisposing causes for decreased milk yield, altered milk composition, and lowered butterfat content in milk [33,34]. Thus, improved mammary gland health may have influenced the greater butterfat and ECM of TRT cows. These outcomes were similar to the findings of previous studies [35] and could be also associated with the improved feeding behavior of cows receiving SCFP [36], attributed mainly to improved rumen functions and VFA production [37]. In this study, we were not able to measure individual DMI, and despite no differences being detected in rumination time, we could not discern any differences associated with feeding behavior that may have occurred between treatments.

### 4.1. SCFP Boosted the Responsiveness of Leukocytes upon Activation in Dairy Cows Irrespective of the SCC Classification at Dry-Off

After dry-off, the mammary gland remodeling elicits a normal inflammatory condition as tissues are repaired [1,2]. During this phase, the acute-phase condition shifts liver protein syntheses in favor of positive acute-phase proteins (i.e., haptoglobin and ceruloplasmin), and the plasma concentrations of other proteins (i.e., albumin, lipoproteins) decrease proportionally to the magnitude and duration of the acute phase [38]. Irrespective of the SCC group, TRT cows had greater concentrations of plasma thiol at −57 DFC, reflecting a heightened endogenous antioxidant availability [39]. In TRT-L cows, greater plasma concentrations of protein detected around dry-off and reduced AGR detected throughout the dry period suggested that the SCFP enhanced the magnitude of systemic inflammatory conditions during early stages of mammary gland remodeling, as AGR reflects the severity of the acute-phase response [3]. Despite that, greater plasma albumin concentrations during the whole dry period were detected in TRT cows irrespective of the SCC group, suggesting that SCFP mitigated the acute-phase response and accelerated homeostasis recovery following the leukocyte activities occurring within the mammary glands at dry-off.

After calving, SCFP supplementation increased the concentrations of haptoglobin and AST and lowered the concentration of Mg in the plasma of TRT cows irrespective of the SCC classification. The latter suggests that the product boosted the acute-phase response of dairy cows following delivery [40]. Such a marked acute-phase response could have transiently impaired liver functions [41] and lowered rumen Mg absorption in TRT cows [42]. This was especially true for cows in the H group, as reflected by the greater concentrations of liver enzymes (GGT and AST) detected in TRT-H cows since −7 DFC. Despite that, the greater plasma Zn of TRT-H cows at 3 DFC suggests that the deleterious side effects associated with the acute-phase response, such as sequestration of plasma Zn by the liver [40], were milder compared to those of the CTR-H cows.

The trends of plasma analytes observed in this study throughout the experimental period were associated with increased hematocrit, red blood cell abundance, and hemoglobin concentration, possibly reflecting a greater oxygen-carrying capacity of the blood in cows that received SCFP [43]. In this context, the heightened inflammatory conditions detected in TRT cows around dry-off and following parturition could depend on a greater capability of the circulating leukocytes to mount a strong and effective inflammatory response upon activation, as circulating oxygen directly affects the efficiency of the respiratory burst [44]. The greater productive performances and lower disease incidence observed in TRT cows suggested that the increased immune responsiveness due to SCFP administration led to smoother adaptation of the cows to the new lactation.

### 4.2. SCFP Lowered the Chronic Low-Grade Systemic Inflammation Affecting Cows with High SCC Classification at Dry-Off

In blood, reduced lymphocyte and greater neutrophil relative abundances, along with the increased RDW observed for H cows before calving, suggest that these cows faced a more severe systemic inflammatory condition during the dry period as compared to L cows. In fact, a greater blood neutrophil-to-lymphocyte ratio was documented as a proxy for systemic inflammation in humans [45]. Furthermore, RDW reflects the heterogeneity of the red blood cell size distribution [46], and increased RDW has been observed in humans under systemic inflammatory conditions [47]. Reduced plasma glucose and cholesterol concentrations measured for H cows compared to L cows before calving support this interpretation. During systemic inflammation, glucose consumption by leukocytes increased tremendously [48]. The acute-phase response affecting the liver could also reduce circulating cholesterol through impairing lipoprotein syntheses [40]. After calving, H cows maintained greater SCC and worse systemic inflammatory conditions (as reflected by greater plasma protein and globulin concentrations). Collectively, these data suggest that increased SCC at dry-off is indictive of chronic low-grade systemic inflammation spanning through the new lactation. Importantly, the microbiological screening performed at dry-off did not record any contagious pathogen growth in the mammary glands of our experimental cows, which dismissed the potential confounding effect of pathogenic bacteria on the metabolic responses measured later in the study, irrespective of the SCC_LV assignment.

Interestingly, ameliorated immune system functions due to SCFP feeding mostly affected the mammary gland remodeling phase in H cows. In plasma, TRT-H cows had lower concentrations of protein, globulin, and ceruloplasmin after dry-off; reduced haptoglobin; and increased albumin and AGR throughout the dry period, reflecting a mitigated inflammatory state. Furthermore, increased thiol availability was observed during the whole dry period in TRT-H cows, possibly accounting for their reduced plasma RFR after dry-off, which reflects an improved redox balance. In this scenario, greater concentrations of circulating leukocytes and lymphocytes detected in TRT-H cows could be consequential to improved leukocyte viability due to an ameliorated redox balance. Therefore, mitigated systemic inflammation and improved redox balance during the dry period can possibly account for the reduced milk SCC of TRT-H cows during the first week of the new lactation, suggesting a positive effect of SCFP in lowering the chronic low-grade inflammatory condition affecting the cows in the H group during the dry period.

### 4.3. A Possible Mode of Action Accounting for Positive SCFP Effects on Improving the Immune Response and the Mammary Gland Health of Dairy Cows

A previous study utilizing a *Streptococcus uberis* mastitis model with mid-lactating cows, including mammary gland biopsies, was conducted to elucidate the possible mode of action behind the beneficial effects of SCFP on udder health [15]. Changes in the expression of several genes consequential to SCFP supplementation in that study align with the trends of plasma analytes detected in the present research. As such, the ameliorated antioxidant availability detected here (e.g., heightened thiol and lowered RFR detected during the dry period) is consistent with the upregulated expression of genes encoding for glutathione metabolism (i.e., *MT3*). The improved leukocyte function found in this study (e.g., stronger inflammation detected around dry-off and calving day) is consistent with the upregulated expression of genes encoding for antimicrobial functions of neutrophils and mammary gland epithelial cells (i.e., *GRO1*, *CSF3*, *CATHL4*, *NOS2*, *IL17C*, *TNF,* and complement coagulation cascade) and with the downregulated expression of several genes encoding for immune-suppressive mediators (i.e., arachidonic acid cascade and steroid hormone biosynthesis). Additionally, changes in whole blood cell count in this study (e.g., greater concentration of circulating leukocytes and lymphocytes detected in TRT-H cows) are consistent with upregulated expression of genes encoding for heat shock proteins (HSPs; i.e., *HSPA6*, *HASPA1A*, *HSPH1*, *DNAJB1*, *BAG3*, and *ZFAND2A*) by SCFP supplementation. The HSPs are mainly involved in reducing apoptosis, improving antigen presentation, and activating lymphocytes [49,50]. Finally, the effectiveness of SCFP in mitigating chronical inflammatory condition in this study (e.g., milder acute phase response affecting TRT-H cows during the dry period and faster resolution of the inflammatory events) was consistent with the upregulated expression of genes encoding for the p21 cascade (i.e., *ATF3* and *IER3*), in that these genes are involved in the resolution of inflammation of tissue by re-establishing homeostasis [51,52].

Among the several concurrent mechanisms involved in modulating inflammation after leukocyte activation, transcription factors such as *Nrf2* exert a ubiquitous effect through several pathways aimed at preventing dysregulated inflammation throughout the body. According to Niture et al. (2014) [53], “*Nrf2* is a transcription factor that regulates the activation of cytoprotective genes that include biotransformation enzymes, antioxidant proteins, antiapoptotic proteins, and proteasomes”, and leukocyte activation serves as the primary stimulus for its expression [54]. The interaction between *Nrf2* and SCFP is consistent with results obtained here and with changes in gene expression documented previously [15], such as upregulated thiol syntheses [55], antioxidant and anti-inflammatory actions [56,57], and upregulated *HSP* expression [58,59,60,61]. Furthermore, the increased blood hematocrit, red blood cell abundance, and hemoglobin concentration found in TRT cows were also consistent with *Nrf2* activation, in that erythropoietic effects have been documented in humans consequentially to the induction of this transcription factor [62]. These outcomes suggest that the SCFP served as an *Nrf2* enhancer when leukocytes activities activated this transcription factor.

Importantly, the results obtained in this study, as well as those of previous experiments conducted on dairy cows, provide a proof of principle of the immune-modulatory effect of SCFP, and the exact mode of action of the product remains unclear. In fact, several active compounds included in the SCFP used in this experiment (i.e., β-glucans, vitamins and antioxidants) have a documented role in improving immune functions and ameliorating udder health through multiple mechanisms. Both active compounds escaping from the rumen degradation and fermentation end-products resulting from alterations induced by SCFP in rumen bacteria populations have the potential to affect the immune systems of the cows through a multitude of pathways, possibly accounting for the positive effect on udder health measured here, as well as for other immune modulatory effects documented in previous studies. Complete elucidation of the active principles responsible for the immunoactivity of SCFP was not possible in this experiment due to trade secrets intended to protect the intellectual property related to the product. Thus, future research should focus on tracking SCFP components post-ingestion to elucidate their bioavailability and specific modes of action on the immune functions of the cows.

## 5. Conclusions

The results from this study suggest the effectiveness of SCFP in improving mammary gland health. Differential effects were detected on systemic inflammation biomarkers and SCC trends among cows with different SCC_LV classifications at dry-off. Supplementing SCFP to cows with increased SCC at the end of lactation would help cows to cope with chronic inflammatory conditions and improve udder health at the onset of the next lactation. Conversely, SCFP supplemented to cows with reduced SCC at the end of lactation would help prevent new intramammary infections from occurring within the first month of lactation. The results suggest that SCFP administration before dry-off could be an effective strategy to improve the mammary health of cows during the dry and early-lactation phases via positively affecting their metabolic adaptation to the peripartum phase.

## Figures and Tables

**Table 1:** Baseline values (i.e., determined 20 days before dry-off) for parity, body condition score, body weight, rumination time, milk yield, milk composition, somatic cell count, estimated milk yield for a 305-d lactation, days in milk, and calving interval of cows included in the study.

Item ^1^	TREAT ^2^	SCC_LV ^3^		Total	*p*-Value		
Unit		L	H		TREAT	SCC_LV	TREAT × SCC_LV
Number	CTR	23	7	30	1.00	1.00	1.00
*n*	TRT	23	7	30			
Parity	CTR	1.78 ± 0.85	2.29 ± 1.38	1.90 ± 0.99	0.89	0.17	0.89
*n*	TRT	1.87 ± 1.06	2.29 ± 1.50	1.97 ± 1.16			
Number by parity							
1	CTR	10	3	13			
	TRT	12	3	15			
2	CTR	9	1	10			
	TRT	4	1	5			
3	CTR	3	1	4			
	TRT	5	2	7			
4	CTR	1	2	3			
	TRT	2	0	2			
5	CTR	0	0	0			
	TRT	0	1	1			
Body status							
Body condition score	CTR	3.02 ± 0.34	2.89 ± 0.32	2.94 ± 0.44	0.89	0.98	0.31
-	TRT	2.91 ± 0.47	3.03 ± 0.47	2.99 ± 0.31			
Body weight *	CTR	780.3 ± 81	734.6 ± 38	762.4 ± 71	0.89	0.88	0.33
kg	TRT	746.7 ± 111	776.9 ± 35	751.9 ± 102			
Productive performances							
Rumination time *	CTR	525.2 ± 72	466.7 ± 101	511.6 ± 83	0.75	0.04	0.36
min/d	TRT	524.9 ± 88	487.5 ± 110	516.1 ± 95			
Milk yield *	CTR	30.8 ± 6.0	26.1 ± 6.6	29.8 ± 6.5	0.61	0.05	0.60
kg/d	TRT	30.4 ± 10	28.3 ± 7.4	29.9 ± 9.5			
Butterfat *	CTR	4.80 ± 0.70	4.63 ± 0.67	4.76 ± 0.70	0.09	0.59	0.22
g/100 g	TRT	4.86 ± 0.69	5.00 ± 0.40	4.89 ± 0.64			
Protein *	CTR	3.34 ± 0.12	3.32 ± 0.12	3.33 ± 0.15	0.57	0.70	0.88
g/100 g	TRT	3.31 ± 0.19	3.36 ± 0.17	3.32 ± 0.19			
Lactose *	CTR	5.00 ± 0.08	4.95 ± 0.08	4.99 ± 0.08	0.89	0.17	0.89
g/100 g	TRT	5.00 ± 0.10	5.00 ± 0.08	5.00 ± 0.10			
Somatic cell count *	CTR	73.7 ± 45	470.4 ± 540	159.9 ± 301	0.58	<0.01	0.53
K/mL	TRT	74.8 ± 44	351.5 ± 495	138.5 ± 266			
Estimated milk yield	CTR	12,267 ± 1425	12,199 ± 996	12,251 ± 1531	0.89	0.37	0.44
kg/305 d	TRT	12,671 ± 2043	11,859 ± 995	12,547 ± 1885			
Reproductive performances							
Days in milk	CTR	285.0 ± 29	282.3 ± 23	284.4 ± 28	0.97	0.61	0.82
d	TRT	287.6 ± 39	280.4 ± 18	286.0 ± 35			
Calving interval	CTR	509.3 ± 180	493.7 ± 186	505.7 ± 170	0.76	0.64	0.84
d	TRT	537.4 ± 186	500.0 ± 176	528.7 ± 178			
Expected calving interval	CTR	365.4 ± 30	361.7 ± 23	366.3 ± 34	0.95	0.55	0.82
d	TRT	368.3 ± 39	360.0 ± 18	364.5 ± 22			

^1^ Parameters marked with * are weekly average values calculated from the individual daily values measured during the baseline week between −83 and −76 days from calving; ^2^ TRT represents cows receiving 19 g/d of *Saccharomyces cerevisiae* fermentation product between −76 and 60 days from calving; CTR represents cows receiving a control diet between −76 and 60 days from calving; ^3^ cows that had an average weekly SCC lower than 100 K/mL milk for primiparous and 200 K/mL milk for multiparous between −83 and −76 days from calving were classified as L, whereas cows that were above those thresholds were classified as H (CTR-L = 23 cows, CTR-H = 7 cows, TRT-L = 23 cows, TRT-H = 7 cows). “*n*” for number and parity; “kg” for body weight; “d” for days in milk, calving interval and expected calving interval.

**Table 2:** Composition (% of dry matter unless otherwise noted) and characteristics of the experimental diets fed during the pre- and postpartum periods.

	Prepartum	Postpartum
Diet ^1^		
DM, kg	12.71	24.13
Corn silage		24.03
Grass silage	23.99	7.89
Alfalfa hay		3.97
Grass hay		3.99
Ryegrass hay	60.13	2.24
High-moisture ear corn	3.87	17.33
Concentrate	10.46	15.05
Linseed cake		3.09
Molasses *		2.42
Sodium bicarbonate		1.22
Hydrogenated fats		1.24
Milking robot plus *		15.91
Mineral vitamin premix	1.55	1.62
Chemical composition		
DM, %	54.8	48.4
CP	12.3	16.8
Soluble CP	4.72	5.17
RDP	8.15	11.2
RUP	4.20	5.54
aNDFom	53.1	30.0
uNDF240	26.0	10.3
Forage NDF	50.9	19.4
Starch	5.02	22.9
Sugar	8.48	6.34
Ether extract	2.58	4.85
Ash	8.14	8.04
Calcium	0.40	0.80
Phosphorous	0.25	0.36
DCAD, meq/100 g	23.7	38.9
NE_L_, mcal/kg	1.34	1.76
NE_L_ allowable milk, kg/d		40.0
AA allowable milk, kd/d		40.1

^1^ Concentrate contained 80% soybean meal (44% crude protein) and 20% roasted soybeans; molasses contained 73.7% DM and 47.1% sugar; milking robot plus consisted of 32% corn, 24.5% soybean meal, 20% wheat bran, 16.2% soyhulls, and 6% mineral and provided 1.3% fat, 12.62% moisture, 21.77% CP, 24.13% NDF, 27.27% starch, 4% fat, and 9.6% ash; lactation mineral vitamin premix contained 14.6% of Ca, 1.7% of P, 16.00% of Na, 3.2% of Mg, 220,000 UI of vitamin A, 70,000 UI of vitamin D3, 600 mg of vitamin E, 5000 mg of niacinamide (vitamin B3), 800 mg of Fe, 350 mg of Cu, 1800 mg of Zn, 1575 mg of Mn, 34 mg of I, and 8 mg of Se; dry mineral vitamin premix contained 13.7% of Ca, 2.5% of P, 6.2% of Na, 8.3% of Mg, 420,000 UI of vitamin A, 210,000 UI of vitamin D3, 6000 mg of vitamin E, 380 mg of vitamin B1, 150 mg of vitamin B6, 1.1 mg of vitamin B12, 2215 mg of Fe, 250 mg of Cu, 5397 mg of Zn, 5500 mg of Mn, 111 mg of I, and 10 mg of Se; parameters marked with * are reported as the average value consumed by the whole herd during the experimental period, as reflected by the automated milking station; DM is dry matter; CP is crude protein; RDP is rumen-degradable protein; RUP is rumen-undegradable protein; aNDFom is amylase corrected-neutral detergent fiber-organic matter; uNFD240 is undegradable neutral detergent fiber after 240 h incubation; DCAD is dietary cation–anion difference; NE_L_ is net energy for lactation; AA is amino acids.

**Table 3:** Incidence of bacterial populations of the mammary gland, assessed at −57 days from calving, in dairy cows with low (L) or high (H) SCC between −83 and −76 days from calving and receiving 19 g/d of *Saccharomyces cerevisiae* fermentation product (TRT) or a control diet (CTR) from −76 to 60 days from calving.

Item ^1^	TREAT ^2^	SCC_LV ^3^		Total
Unit		L	H	
Animals’ distribution				
Culture-negative cows	CTR	9	1	10
*n*	TRT	6	2	8
Culture-positive cows	CTR	10	3	13
*n*	TRT	7	4	11
Cows affected by multiple pathogens	CTR	0	0	0
*n*	TRT	2	1	3
Pathogens				
*Staphylococcus* spp.	CTR	5	2	7
*n*	TRT	4	3	7
*Bacillus* spp.	CTR	0	0	0
*n*	TRT	1	0	1
*Streptococcus dysagalactiae*	CTR	1	0	1
*n*	TRT	1	0	1
*Streptococcus uberis*	CTR	4	0	4
*n*	TRT	2	1	3
*Escherichia coli*	CTR	0	1	1
*n*	TRT	0	1	1
Yeasts	CTR	0	0	0
*n*	TRT	1	0	1
Non-Pathogens				
Mixed bacterial growth	CTR	7	4	11
*n*	TRT	10	2	12

^1^ Culture-negative cows showed no signs of bacterial growth (pathogens or not) in any quarter; culture-positive cows had at least 1 quarter affected by pathogen species; cows affected by multiple pathogens had more than one pathogen species in the same or in different quarters; ^2^ TRT represents cows receiving 19 g/d of *Saccharomyces cerevisiae* fermentation product between −76 and 60 days from calving; CTR represents cows receiving a control diet between −76 and 60 days from calving; ^3^ Cows that had an average weekly SCC lower than 100 K/mL milk for primiparous and 200 K/mL milk for multiparous cows between −83 and −76 days from calving were classified as L, whereas cows that were above those thresholds were classified as H (CTR-L = 23 cows, CTR-H = 7 cows, TRT-L = 23 cows, TRT-H = 7 cows).

**Table 4:** Disease incidence in dairy cows with low (L) or high (H) SCC between −83 and −76 days from calving and receiving 19 g/d of *Saccharomyces cerevisiae* fermentation product (TRT) or a control diet (CTR) from −76 to 60 days from calving.

Item ^1^	TREAT ^2^	SCC_LV ^3^		Total
Unit		L	H	
Animals’ distribution				
Healthy cows	CTR	8	2	10
*n*	TRT	15	4	19
*p*-value		0.04	0.28	0.02
Multiple	CTR	2	0	2
*n*	TRT	1	0	1
*p*-value		0.55	1.00	0.55
Diseases				
Metritis	CTR	1	1	2
*n*	TRT	0	0	0
*p*-value		0.31	0.30	0.55
Pyometra	CTR	3	1	4
*n*	TRT	2	0	2
*p*-value		0.63	0.30	0.39
Mastitis	CTR	8	1	9
*n*	TRT	1	1	2
*p*-value		<0.01	1.00	0.02
Milk fever	CTR	1	0	1
*n*	TRT	1	0	1
*p*-value		1.00	1.00	1.00
Ketosis	CTR	1	0	1
*n*	TRT	0	0	0
*p*-value		0.31	1.00	0.31
Retained placenta	CTR	3	1	4
*n*	TRT	3	1	4
*p*-value		1.00	1.00	1.00

^1^ Healthy cows did not have any clinical diseases during the whole experimental period; cows that had more than one clinical problem during the experimental period were designated as “Multiple”; ^2^ TRT represents cows receiving 19 g/d of *Saccharomyces cerevisiae* fermentation product between −76 and 60 days from calving; CTR represents cows receiving a control diet between −76 and 60 days from calving; ^3^ Cows that had an average weekly SCC lower than 100 K/mL milk for primiparous and 200 K/mL milk for multiparous cows between −83 and −76 days from calving were classified as L, whereas cows that were above those thresholds were classified as H (CTR-L = 23 cows, CTR-H = 7 cows, TRT-L = 23 cows, TRT-H = 7 cows).

**Table 5:** Body condition score, rumination time, milk yield, energy-corrected milk, milk quality biomarkers, and SCC of milk in dairy cows with low (L) or high (H) SCC between −83 and −76 days from calving and receiving 19 g/d of *Saccharomyces cerevisiae* fermentation product (TRT) or a control diet (CTR) from −76 to 60 days from calving.

Item ^1^	TREAT ^2^	Prepartum			Postpartum			SEM ^4^	Period ^5^	*p*-Value			
		SCC_LV ^3^		Total	SCC_LV ^3^		Total						
Unit		L	H		L	H				TREAT	TIME	SCC_LV	TREAT × SCC_LV
BCS	CTR	3.05	3.00	3.04	2.92	2.89	2.91	0.11	Prepartum	0.98	<0.01	0.86	0.42
-	TRT	2.99	3.07	3.01	2.84	2.95	2.87	0.11	Postpartum	0.87	<0.01	0.59	0.34
	Tot	3.02	3.04		2.88	2.92							
Rumination	CTR	528.4	514.0	525.0	541.2	513.0	534.6	21.8	Prepartum	0.80	<0.01	0.37	1.00
min/d	TRT	533.8	516.9	529.8	533.7	513.6	529.0	21.8	Postpartum	0.72	<0.01	0.19	0.97
	Total	531.1	515.4		537.4	513.3		21.7					
Milk yield	CTR				44.51	42.58	44.06	3.06	Prepartum				
kg/d	TRT				46.84	41.22	45.53	3.06	Postpartum	1.00	<0.01	0.15	0.51
	Total				45.67	41.90							
ECM	CTR				46.20	44.69	45.85	3.21	Prepartum				
kg/d	TRT				50.17	44.96	48.95	3.21	Postpartum	0.57	<0.01	0.21	0.51
	Total				48.19	44.82							
Butterfat	CTR				3.89	3.90	3.89	0.19	Prepartum				
g/100 g	TRT				4.17	4.29	4.20	0.19	Postpartum	0.03	<0.01	0.66	0.75
	Total				4.03	4.10							
Protein	CTR				3.43	3.46	3.44	0.08	Prepartum				
g/100 g	TRT				3.44	3.46	3.45	0.08	Postpartum	0.96	<0.01	0.81	0.90
	Total				3.44	3.46							
Lactose	CTR				4.93	4.89	4.92	0.03	Prepartum				
g/100 g	TRT				4.91	4.92	4.91	0.03	Postpartum	0.65	<0.01	0.64	0.27
	Total				4.92	4.90							
SCC	CTR				3.01	3.47	3.11	0.59	Prepartum				
SCS	TRT				2.36	3.37	2.60	0.59	Postpartum	0.25	<0.01	0.10	0.88
	Total				2.68	3.42							

^1^ BCS is body condition score; ECM is energy-corrected milk = [milk yield (kg/d) × (0.383 × butterfat (g/100 g) + 0.242 × protein (g/100 g) + 0.1571 × lactose (g/100 g) + 0.207)/3.14]; SCS = somatic cell score calculated as Log2(SCC/100) + 3; ^2^ TRT represents cows receiving 19 g/d of *Saccharomyces cerevisiae* fermentation product between −76 and 60 days from calving; CTR represents cows receiving a control diet between −76 and 60 days from calving. ^3^ Cows that had an average weekly SCC lower than 100 K/mL milk for primiparous and 200 K/mL milk for multiparous cows between −83 and −76 days from calving were classified as L, whereas cows that were above those thresholds were classified as H (CTR-L = 23 cows, CTR-H = 7 cows, TRT-L = 23 cows, TRT-H = 7 cows). ^4^ Standard error  =  largest standard error for the fixed effects. ^5^ Prepartum considered −57, −54, −44, and −7 DFC measures for BCS and phases 1 (i.e., from −55 to −35 DFC) and 2 (i.e., from −14 to 0 DFC) measures for rumination time. Postpartum considered 3, 14, 28, and 60 DFC for BCS and phases 3 (i.e., from 1 to 7 DFC), 4 (i.e., from 8 to 21 DFC), 5 (i.e., from 22 to 40 DFC), and 6 (i.e., from 41 to 60 DFC) measures for rumination time, milk yield, ECM, milk composition, and SCC.

**Table 6:** Plasma analytes reflecting energy, protein, and mineral metabolism; liver function; inflammation; and redox balance in dairy cows with low (L) or high (H) SCC between −27 and −20 days from dry-off and receiving 19 g/d of *Saccharomyces cerevisiae* fermentation product (TRT) or a control diet (CTR) from −20 days from dry-off to 60 days from calving.

Item ^1^	TREAT ^2^	Prepartum			Postpartum			SEM ^4^	Period ^5^	*p*-Value			
		SCC_LV ^3^		Total	SCC_LV ^3^		Total						
Unit		L	H		L	H				TREAT	TIME	SCC_LV	TREAT × SCC_LV ^6^
Glucose	CTR	4.42	4.31	4.40	3.97	3.87	3.94	0.13	Prepartum	0.78	<0.01	0.03	0.79
mmol/L	TRT	4.42	4.28	4.39	3.90	3.83	3.88	0.13	Postpartum	0.60	<0.01	0.43	0.92
	Total	4.42	4.30		3.93	3.85							
Urea	CTR	4.55	4.63	4.57	4.51	4.93 ^a^	4.61	0.29	Prepartum	0.45	0.01	0.20	0.40
mmol/L	TRT	4.53	4.94	4.63	4.59	4.13 ^b^	4.48	0.29	Postpartum	0.13	<0.01	0.90	0.07
	Total	4.54	4.79		4.55	4.52							
Magnesium	CTR	0.95	0.92	0.94	0.98	1.04 ^A^	0.99	0.04	Prepartum	0.90	<0.01	0.08	0.69
mmol/L	TRT	0.97	0.91	0.95	0.96	0.87 ^B^	0.94	0.04	Postpartum	<0.01	<0.01	0.67	0.02
	Total	0.96	0.92		0.97	0.96							
AST	CTR	112.5	127.3	115.9	99.0	117.0 ^a^	103.2	10.1	Prepartum	0.56	<0.01	0.59	0.57
U/L	TRT	117.1	121.2	118.0	106.0	148.8 ^b^	116.0	9.6	Postpartum	<0.01	<0.01	0.15	<0.01
	Total	114.8	124.2		102.5	132.9							
Protein	CTR	79.40 ^a^	83.62	80.38	74.64	79.57	75.79	1.58	Prepartum	1.00	<0.01	0.10	0.07
g/L	TRT	81.59 ^b^	81.43	81.55	76.04	78.73	76.67	1.58	Postpartum	0.83	<0.01	<0.01	0.38
	Total	80.50	82.52		75.34	79.15							
Globulin	CTR	42.06 ^a^	47.43 ^a^	43.32	38.72	42.12	39.52	1.79	Prepartum	0.52	<0.01	0.12	0.02
g/L	TRT	44.39 ^b^	43.32 ^b^	44.14	40.29	42.38	40.78	1.79	Postpartum	0.53	<0.01	0.06	0.65
	Total	43.23	45.38		39.51	42.25							
Haptoglobin	CTR	0.11	0.23 ^a^	0.14	0.26	0.23	0.25	0.06	Prepartum	0.16	0.39	0.34	0.01
g/L	TRT	0.15	0.09 ^b^	0.14	0.31	0.36	0.32	0.06	Postpartum	0.05	<0.01	0.77	0.32
	Total	0.13	0.16		0.28	0.30							
Albumin	CTR	37.33	36.19 ^a^	37.07	35.92	36.59	36.07	0.71	Prepartum	0.05	<0.01	0.78	0.02
g/L	TRT	37.21	38.10 ^b^	37.42	35.75	36.35	35.89	0.71	Postpartum	0.73	<0.01	0.27	0.95
	Total	37.27	37.15		35.84	36.47							
Cholesterol	CTR	5.14	4.81	5.07	3.18	3.47	3.25	0.32	Prepartum	0.29	<0.01	0.07	0.61
mmol/L	TRT	5.00	4.41	4.86	3.25	3.27	3.25	0.32	Postpartum	0.80	<0.01	0.55	0.61
	Total	5.07	4.61		3.22	3.37							
AGR	CTR	0.91 *^a^*	0.79 ^a^	0.88	0.95	0.91	0.94	0.05	Prepartum	0.19	<0.01	0.45	<0.01
-	TRT	0.86 *^b^*	0.93 ^b^	0.87	0.91	0.90	0.91	0.05	Postpartum	0.54	<0.01	0.50	0.70
	Total	0.88	0.86		0.93	0.90							
Thiol group	CTR	289.0	279.0 ^a^	286.6	293.9	303.4	296.1	11.8	Prepartum	0.04	<0.01	0.39	0.04
µmol/L	TRT	288.7	312.5 ^b^	294.3	287.8	309.8	292.9	11.8	Postpartum	0.99	0.02	0.10	0.51
	Total	288.9	295.7		290.8	306.6							

^1^ AST is glutamate oxalacetate transaminase; AGR is albumin to globulin ratio = Albumin (g/L)/ Globulin (g/L); ^2^ TRT represents cows receiving 19 g/d of *Saccharomyces cerevisiae* fermentation product −20 days from dry-off and 60 days from calving; CTR represents cows receiving a control diet between −20 days from dry-off and 60 days from calving. ^3^ Cows that had an average weekly SCC lower than 100 K/mL milk for primiparous and 200 K/mL milk for multiparous cows between −27 and −20 days from dry-off were classified as L, whereas cows that were above those thresholds were classified as H (CTR-L = 23 cows, CTR-H = 7 cows, TRT-L = 23 cows, TRT-H = 7 cows). ^4^ Standard error  =  largest standard error for the fixed effects. ^5^ Prepartum considered −57, −54, −44, and −7 DFC samples. Postpartum considered 3, 14, and 28 DFC samples. ^6 A/B^ is *p* < 0.01; ^a/b^ is *p* < 0.05 and *^a/b^* is *p* < 0.1 for differences among means within a column. Letters are only presented when the interaction effect is significant.

**Table 7:** Complete blood cell counts in dairy cows with low (L) or high (H) SCC between −27 and −20 days from dry-off and receiving 19 g/d of *Saccharomyces cerevisiae* fermentation product (TRT) or a control diet (CTR) from −20 days from dry-off to 60 days from calving.

Item ^1^	TREAT ^2^	Prepartum			Postpartum			SEM ^4^	Period ^5^	*p*-Value			
		SCC_LV ^3^		Total	SCC_LV ^3^		Total						
Unit		L	H		L	H				TREAT	TIME	SCC_LV	TREAT × SCC_LV ^6^
RBC	CTR	6.73	6.57	6.69	5.85	5.79	5.84	0.19	Prepartum	0.01	<0.01	0.44	0.71
M/µL	TRT	7.02	6.97	7.01	5.92	6.01	5.94	0.19	Postpartum	0.33	<0.01	0.94	0.60
	Total	6.87	6.77		5.89	5.90							
Hemoglobin	CTR	11.62	11.25	11.54	10.02	9.92 ^a^	9.99	0.25	Prepartum	0.01	<0.01	0.81	0.11
g/dL	TRT	11.86	12.14	11.92	10.02	10.70 ^b^	10.18	0.25	Postpartum	0.06	<0.01	0.15	0.05
	Total	11.74	11.69		10.02	10.31							
RDW	CTR	18.91	20.69	19.32	18.01	18.76	18.18	0.62	Prepartum	0.64	0.01	0.08	0.08
%	TRT	19.56	19.56	19.56	18.63	18.63	18.63	0.62	Postpartum	0.55	<0.01	0.37	0.37
	Total	19.23	20.13		18.32	18.69							
Hematocrit	CTR	34.98	33.68 ^a^	34.68	29.93	29.73	29.88	0.95	Prepartum	0.02	<0.01	0.67	0.10
M/µL	TRT	35.40	36.17 ^b^	35.58	29.96	31.55	30.34	0.95	Postpartum	0.23	<0.01	0.37	0.25
	Total	35.19	34.93		29.95	30.64							
Monocytes	CTR	0.92	0.82	0.90	1.23 ^a^	0.91	1.16	0.09	Prepartum	0.41	0.03	0.86	0.14
K/µL	TRT	0.88	0.96	0.90	1.09 ^b^	1.07	1.09	0.09	Postpartum	0.91	<0.01	0.03	0.05
	Total	0.90	0.89		1.16	0.99							
Basophils	CTR	0.06	0.05	0.06	0.06 *^a^*	0.04	0.05	0.01	Prepartum	0.28	0.02	0.43	0.29
K/µL	TRT	0.06	0.07	0.06	0.05 *^b^*	0.05	0.05	0.01	Postpartum	0.73	0.79	0.37	0.04
	Total	0.06	0.06		0.05	0.05							
Basophils	CTR	0.83	0.81	0.82	0.87 ^a^	0.62	0.82	0.12	Prepartum	0.66	0.01	0.87	0.64
%WBC	TRT	0.82	0.87	0.83	0.67 ^b^	0.81	0.71	0.12	Postpartum	0.96	0.92	0.56	0.04
	Total	0.83	0.84		0.77	0.72							
Neutrophils	CTR	44.98	49.85	46.12	43.59	44.27	43.75	3.32	Prepartum	0.84	<0.01	0.02	0.49
%WBC	TRT	46.11	49.04	46.80	42.25	46.67	43.28	3.32	Postpartum	0.24	0.01	0.35	0.43
	Total	45.55	49.45		42.92	45.47							
Lymphocytes	CTR	2.90	2.34	2.77	2.62	2.35	2.56	0.30	Prepartum	0.24	0.01	0.04	0.43
K/µL	TRT	2.98	2.72	2.92	2.62	2.47	2.59	0.30	Postpartum	0.81	0.18	0.38	0.81
	Total	2.94	2.53		2.62	2.41							
Lymphocytes	CTR	36.93	33.00	36.01	36.85	37.80	37.07	2.76	Prepartum	0.52	0.01	0.02	0.73
%WBC	TRT	37.34	34.44	36.67	38.57	35.95	37.96	2.76	Postpartum	0.98	0.86	0.71	0.43
	Total	37.13	33.72		37.71	36.87							

^1^ RBC is red blood cells; RDW is red blood cell distribution wide; and WBC is white blood cells. ^2^ TRT represents cows receiving 19 g/d of *Saccharomyces cerevisiae* fermentation product between −20 days from dry-off and 60 days from calving; CTR represents cows receiving a control diet between −20 days from dry-off and 60 days from calving. ^3^ Cows that had an average weekly SCC lower than 100 K/mL milk for primiparous and 200 K/mL milk for multiparous cows between −27 and −20 days from dry-off were classified as L, whereas cows that were above those thresholds were classified as H (CTR-L = 23 cows, CTR-H = 7 cows, TRT-L = 23 cows, TRT-H = 7 cows). ^4^ Standard error  =  largest standard error for the fixed effects. ^5^ Prepartum considered −57, −54, −44, and −7 DFC samples. Postpartum considered 3, 14, and 28 DFC samples. ^6 a/b^ is *p* < 0.05 and *^a/b^* is *p* < 0.1 for differences among means within a column. Letters are only presented when the interaction effect is significant.

## Data Availability

Data used in this study are available upon request to the corresponding author.

## Supplementary Materials

The following supporting information can be downloaded at: https://www.mdpi.com/article/10.3390/ani15040480/s1, Table S1: Automatic milking system supplement schedule based on DIM and milk yield; Table S2: Intra- and inter-assay coefficient of variations, limit of quantification (LOQ), codes of commercial kits used, calibrators and quality controls used for plasma analytes included in the study; Table S3: Plasma analytes reflecting energy, protein and mineral metabolism, liver function, inflammation, and redox balance in dairy cows having a low (L) or high (H) SCC between −83 and −76 days from calving and receiving 19 g/d of *Saccharomyces cerevisiae* fermentation product (TRT) or a control diet (CTR) from −76 to 60 days from calving; Table S4: Complete blood count in dairy cows with low (L) or high (H) SCC between −83 and −76 days from calving and receiving 19 g/d of *Saccharomyces cerevisiae* fermentation product (TRT) or a control diet (CTR) from −76 to 60 days from calving.
