# Supplementary material for: Impact of a Saccharomyces cerevisiae Fermentation Product Supplemented from 20 Days Before Dry-Off Through 60 Days of Lactation on the Metabolic Adaptation of Dairy Cows to the Peripartum Phase
Source: Animals (Basel). 2025 Feb 8;15(4):480. doi: 10.3390/ani15040480 (PMC11851521; doi:10.3390/ani15040480)
Supplement: Supplementary file 1 [file animals-15-00480-s001.zip › animals-3352794-supplementary.pdf]

Table S1. Automatic milking system supplement schedule based on DIM and milk yield.

DIM	Milk yield (kg/d)	Milking robot plus[1] (kg/d)		Molasse[2] (kg/d)
		Primiparous	Multiparous	
0		3	3	0.1
14		5	5	0.5
15-60	0	4	4	0.2
	30	5	5	0.2
	35	5	5	0.3
	40	5	5.5	0.4
	45	5.5	6	0.4
Days prior to dry-off				
21		2	2	0
1		2	2	0

[1]Product consisted of 32% corn, 24.5% soybean meal, 20% wheat bran, 16.2% soyhulls, and 6% mineral and provided 1.3% fat, 12.62% moisture, 21.77% CP, 24.13% NDF, 27.27% starch, 4% fat, and 9.6% ash.
[2]73.7% DM, 47.1% sugar.

Table S2. Intra- and inter-assay coefficient of variations, limit of quantification (LOQ), codes of commercial kits used, calibrators and quality controls used for plasma analytes included in the study.

<table>
<tr><th>Parameter, UM10 [9]</th><th>Intra</th><th>Inter</th><th>LOQ</th><th>Kit</th><th>Calibrator</th><th>Quality control</th></tr>
<tr><td>Glucose, mmol/L</td><td>0.67</td><td>1.05</td><td>0.1</td><td>18250840[1]</td><td>Homemade bovine standard</td><td>Homemade bovine standard; SeraChem Control Level 1, 00181624121; Bov Asy Control 2, AN10263</td></tr>
<tr><td>Insulin</td><td>3.26</td><td>5.67</td><td>0.2</td><td>10-1201-01 Insulin ELISA Bovine[2]</td><td></td><td>10-1221-01 Control Animal Insulin (3x0.5 ml)</td></tr>
<tr><td>NEFA, mmol/L</td><td>0.85</td><td>2.14</td><td>0.01</td><td>NEFA-HR(2) R1 Set, 434-91795[3]</td><td>NEFA standard, 270-77000[3]</td><td rowspan="12">Homemade bovine standard; SeraChem Control Level 1, 00181624121; Bov Asy Control 2, AN10263</td></tr>
<tr><td>BHB, mmol/L</td><td>0.96</td><td>1.71</td><td>0.1</td><td>RB1007[4]</td><td>BHB standard included in the kit</td></tr>
<tr><td>Urea, mmol/L</td><td>0.68</td><td>1.97</td><td>0.01</td><td>18255440[1]</td><td rowspan="4">Homemade bovine standard</td></tr>
<tr><td>Ca, mmol/L</td><td>1.15</td><td>1.59</td><td>0.2</td><td>18164340[1]</td></tr>
<tr><td>P, mmol/L</td><td>0.26</td><td>0.82</td><td>0.01</td><td>18251240[1]</td></tr>
<tr><td>Mg, mmol/L</td><td>0.52</td><td>1.51</td><td>0.02</td><td>18164540[1]</td></tr>
<tr><td>Zn, mmol/L</td><td>1.13</td><td>1.99</td><td>0.5</td><td>CL100S[3]</td><td>Bov Asy Control 2[5]</td></tr>
<tr><td>Creatinine, µmol/L</td><td>0.93</td><td>1.51</td><td>18</td><td>18255540[1]</td><td>Homemade bovine standard</td></tr>
<tr><td>Bilirubin, µmol/L</td><td>0.70</td><td>1.75</td><td>2</td><td>18254640[1]</td><td>Bov Asy Control 2[5]</td></tr>
<tr><td>AST, U/L</td><td>1.55</td><td>1.66</td><td>1</td><td>18257540[1]</td><td>No calibration is required</td></tr>
<tr><td>GGT, U/L</td><td>0.95</td><td>2.33</td><td>2</td><td>18257640[1]</td><td>No calibration is required</td></tr>
<tr><td>ALP, U/L</td><td>0.76</td><td>1.76</td><td>2</td><td>18259640[1]</td><td>ReferrIL E, 0018256300[1]</td></tr>
<tr><td>Myeloperoxidase, U/L</td><td>0.82</td><td>2.22</td><td>10</td><td>-</td><td>No calibration is required</td><td>Homemade bovine standard; SeraChem Control Level 1, 00181624121</td></tr>
<tr><td>Total protein, g/L</td><td>0.47</td><td>1.20</td><td>10</td><td>18251440[1]</td><td>Homemade bovine standard</td><td rowspan="5">Homemade bovine standard; SeraChem Control Level 1, 00181624121; Bov Asy Control 2, AN10263</td></tr>
<tr><td>Haptoglobin, g/L</td><td>1.50</td><td>2.74</td><td>0.01</td><td>-</td><td>No calibration is required</td></tr>
<tr><td>Ceruloplasmin, µmol/L</td><td>0.50</td><td>2.36</td><td>0.1</td><td>-</td><td>Human plasma ceruloplasmin, A50143H[6]</td></tr>
<tr><td>Albumin, g/L</td><td>0.39</td><td>1.04</td><td>16</td><td>18250040[1]</td><td>Homemade bovine standard</td></tr>
<tr><td>Cholesterol, mmol/L</td><td>1.56</td><td>1.38</td><td>0.1</td><td>182505401</td><td>Homemade bovine standard</td></tr>
<tr><td>Retinol, µmol/L</td><td>2.72</td><td>2.70</td><td>-</td><td>-</td><td>R763[3]</td><td>Homemade bovine standard;</td></tr>
<tr><td>Paraoxonase, U/mL</td><td>1.38</td><td>4.60</td><td>1.2</td><td>-</td><td>No calibration is required</td><td>Homemade bovine standard; SeraChem Control Level 1, 00181624121; Bov Asy Control 2, AN10263</td></tr>
<tr><td>Tocopherol, µmol/L</td><td>3.38</td><td>3.52</td><td>-</td><td>-</td><td>T325[1]</td><td rowspan="2">Homemade bovine standard;</td></tr>
<tr><td>β-Carotene, µmol/L</td><td>5.55</td><td>6.32</td><td>-</td><td>-</td><td>C458[3]</td></tr>
</table>

FRAP, mmol/L	1.46	2.79	30	-	TROLOX, 238813-1G[7]	Homemade bovine standard; SeraChem Control Level 1, 00181624121; Bov Asy Control 2, AN10263; TROLOX, 238813-1G8
Thiol groups, mmol/L	0.55	2.35	3.5	MC433[5]	Calibrator SHp, MC.030[8]	Homemade bovine standard; SeraChem Control Level 1, 00181624121; Bov Asy Control 2, AN10263 Calibrator SHp, MC.0309
ROMt, mg H2O2/dL	1.28	1.63	3.2	MC003[5]	Calibrator d-ROMs, MC.030[8]	Homemade bovine standard SeraChem Control Level 1, 00181624121; Control Serum, MC0314; Calibrator d-ROMs, MC0304

[1]Instrumentation Laboratory Werfen, Milano, Italy.
[2]D.B.A. Italia s.r.l., Via Umbria, 10 20090 Segrate (MI) Italy
[3]Wako Chemicals GmbH, Neuss, Germany.
[4]Randox Laboratories Ltd., Crumlin, County Antrim, UK.
[5]Diacron International S.r.l., Grosseto, Italy.
[6]Meridian Life Science, Memphis, USA.
[7]Sigma-Aldrich Chemie GmbH, Steinheim, Germany.
[8]Carlo Erba, Rodano, Milano, Italy.
[9]NEFA = non-esterified fatty acids, BHB = β-Hydroxybutyrate; AST = aspartate aminotransferase; GGT = γ-glutamyl transferase; ALP = alkaline phosphatase; FRAP = ferric reducing antioxidant power, ROMt = total reactive oxygen metabolites.

Table S3. Plasma analytes reflecting energy, protein and mineral metabolism, liver function, inflammation and redox balance in dairy cows having a low (L) or high (H) SCC between -83 and -76 days from calving and receiving 19 g/d of *Saccharomyces cerevisiae* fermentation product (TRT) or a control diet (CTR) from -76 thru 60 days from calving.

Item	TREAT [2]	Prepartum			Postpartum			SEM [4]	Period	P-VALUE			
		SCC_LV [3]		Tot	SCC_LV		Tot						
Unit [1]		L	H		L	H				TREAT	TIME	SCC_LV	TREAT X SCC_LV
Insulin	CTR	9.62	10.28	9.78	6.07	4.89	5.80	1.52	Prepartum	0.37	<0.01	0.97	0.57
µmol/L	TRT	11.43	10.68	11.25	4.77	6.79	5.24	1.52	Postpartum	0.78	<0.01	0.69	0.13
	Tot	10.52	10.48		5.42	5.84							
NEFA	CTR	0.23	0.28	0.24	0.66	0.69	0.66	0.11	Prepartum	0.95	<0.01	0.41	0.68
mmol/L	TRT	0.25	0.26	0.25	0.69	0.70	0.69	0.11	Postpartum	0.78	<0.01	0.83	0.91
	Tot	0.24	0.27		0.67	0.69							
BHB	CTR	0.40	0.41	0.40	0.63	0.67	0.64	0.21	Prepartum	0.52	<0.01	0.53	0.85
mmol/L	TRT	0.41	0.43	0.42	0.74	0.79	0.75	0.21	Postpartum	0.49	0.04	0.79	0.96
	Tot	0.41	0.42		0.69	0.73							
NAR	CTR	0.01	0.01	0.01	0.02	0.02	0.02	0.003	Prepartum	0.82	<0.01	0.32	0.56
-	TRT	0.01	0.01	0.01	0.02	0.02	0.02	0.003	Postpartum	0.74	<0.01	0.88	0.90
	Tot	0.01	0.01		0.02	0.02							
QUICKI	CTR	0.36	0.36	0.36	0.53	0.44	0.51	0.12	Prepartum	0.63	<0.01	0.75	0.74
-	TRT	0.36	0.36	0.36	0.45	0.43	0.45	0.12	Postpartum	0.66	<0.01	0.56	0.74
	Tot	0.36	0.36		0.49	0.43							
rQUICKI	CTR	0.49	0.47	0.49	0.39	0.49	0.41	0.10	Prepartum	0.84	<0.01	0.44	0.50
-	TRT	0.49	0.48	0.49	0.50	0.48	0.50	0.10	Postpartum	0.51	0.02	0.64	0.47
	Tot	0.49	0.48		0.44	0.48							
rQUICKIbhb	CTR	0.65	0.59	0.63	0.52	0.56	0.53	0.05	Prepartum	0.99	<0.01	0.39	0.51
-	TRT	0.62	0.61	0.62	0.58	0.57	0.58	0.05	Postpartum	0.34	0.04	0.64	0.50
	Tot	0.63	0.60		0.55	0.57							
Creatinine	CTR	91.04	89.39	90.65	89.29	90.57	89.59	2.88	Prepartum	0.45	<0.01	0.76	0.68
µmol/L	TRT	91.82	92.05	91.87	88.23	90.98	88.87	2.88	Postpartum	0.89	<0.01	0.39	0.75
	Tot	91.43	90.72		88.76	90.78							
Calcium	CTR	2.58	2.58	2.58	2.29	2.32	2.30	0.07	Prepartum	0.69	<0.01	0.27	0.30
mmol/L	TRT	2.56	2.61	2.57	2.25	2.31	2.26	0.07	Postpartum	0.65	<0.01	0.47	0.78
	Tot	2.57	2.60		2.27	2.31							
Phosphorus	CTR	2.23	2.12	2.19	1.50	1.41	1.48	0.10	Prepartum	0.85	<0.01	0.35	0.67

mmol/L	TRT	2.17	2.14	2.16	1.52	1.48	1.51	0.10	Postpartum	0.55	0.35	0.40	0.74
	Tot	2.19	2.13		1.51	1.44							
Zinc	CTR	10.95	10.59	10.86	6.75	7.50	6.93	0.78	Prepartum	0.39	<0.01	0.45	0.19
µmol/L	TRT	10.65	11.97	10.96	6.80	7.24	6.91	0.78	Postpartum	0.87	<0.01	0.33	0.80
	Tot	10.80	11.28		6.78	7.37							
Bilirubin	CTR	1.11	1.50	1.20	4.53	5.81	4.83	0.91	Prepartum	0.65	<0.01	0.83	0.14
µmol/L	TRT	1.34	1.06	1.28	4.92	5.05	4.95	0.91	Postpartum	0.80	<0.01	0.34	0.44
	Tot	1.23	1.28		4.72	5.43							
GGT	CTR	33.18	26.52	31.63	22.98	22.38	22.84	3.14	Prepartum	0.85	<0.01	0.16	0.24
U/L	TRT	30.61	30.03	30.48	24.31	26.32	24.78	3.14	Postpartum	0.23	<0.01	0.75	0.55
	Tot	31.90	28.28		23.65	24.35							
ALP	CTR	56.01	50.30	54.68	44.41	48.71	45.41	6.48	Prepartum	0.92	<0.01	0.34	0.90
U/L	TRT	55.82	51.50	54.81	43.64	47.71	44.59	6.48	Postpartum	0.81	0.01	0.26	0.97
	Tot	55.92	50.90		44.03	48.21							
Myeloperoxidase	CTR	395.2	394.8	395.1	388.5	387.3	388.3	12.3	Prepartum	0.38	0.25	0.15	0.13
U/L	TRT	389.3	416.8	395.7	392.9	382.2	390.4	12.3	Postpartum	0.97	<0.01	0.55	0.63
	Tot	392.3	405.8		390.7	384.7							
Interleukin-6	CTR	1055.9	1077.8	1061.0	619.4	656.8	628.2	417.2	Prepartum	0.51	0.76	0.85	0.89
pg/mL	TRT	668.6	807.9	701.1	607.6	356.0	548.9	417.2	Postpartum	0.63	0.46	0.73	0.66
	Tot	862.3	942.8		613.5	506.4							
Ceruloplasmin	CTR	2.44	2.57	2.47	2.72	2.64	2.70	0.16	Prepartum	0.43	<0.01	0.87	0.14
g/L	TRT	2.51	2.34	2.47	2.66	2.80	2.69	0.16	Postpartum	0.69	0.16	0.82	0.41
	Tot	2.47	2.46		2.69	2.72							
Retinol	CTR	27.99	27.30	27.83	20.57	23.10	21.16	2.21	Prepartum	0.30	<0.01	0.55	0.31
U/mL	TRT	28.04	30.76	28.67	21.48	20.93	21.35	2.21	Postpartum	0.73	<0.01	0.58	0.39
	Tot	28.01	29.03		21.02	22.01							
Paraoxonase	CTR	86.90	90.50	87.74	77.56	81.24	78.42	5.83	Prepartum	0.52	<0.01	0.71	0.20
U/mL	TRT	89.47	82.88	87.93	76.30	74.31	75.84	5.83	Postpartum	0.39	<0.01	0.86	0.55
	Tot	88.19	86.69		76.93	77.77							
MPR	CTR	4.65	4.58	4.64	5.22	5.11	5.20	0.58	Prepartum	0.45	<0.01	0.17	0.10
-	TRT	4.44	5.14	4.60	5.62	5.84	5.67	0.58	Postpartum	0.23	<0.01	0.91	0.72
	Tot	4.55	4.86		5.42	5.48							
LFI	CTR				0.78	0.97	0.82	0.97	Postpartum	0.80	-	0.72	0.90
-	TRT				0.48	0.87	0.57	0.87					
	Tot				0.63	0.92							
ROMt	CTR	13.35	13.67	13.42	13.87	13.24	13.72	0.69	Prepartum	0.37	<0.01	0.80	0.33

mg H2O2/100 mL	TRT	13.38	12.84	13.25	13.33	13.88	13.46	0.69	Postpartum	0.93	<0.01	0.94	0.30
	Tot	13.36	13.25		13.60	13.56							
GPx	CTR	64.47	60.04	63.43	51.66	47.56	50.70	3.67	Prepartum	0.56	<0.01	0.58	0.35
U/L	TRT	63.43	64.54	63.69	51.03	50.89	51.00	3.67	Postpartum	0.62	<0.01	0.44	0.47
	Tot	63.95	62.29		51.34	49.22							
FRAP	CTR	145.9	140.8	144.7	156.7	157.8	157.0	5.2	Prepartum	0.55	<0.01	0.67	0.15
µmol/L	TRT	143.5	146.3	144.2	155.1	154.1	154.8	5.2	Postpartum	0.53	0.12	0.99	0.81
	Tot	144.7	143.5		155.9	156.0							
Tocopherol	CTR	5.61	5.22	5.51	3.29	3.83	3.42	0.42	Prepartum	0.38	<0.01	0.24	0.91
U/mL	TRT	5.31	4.99	5.23	3.87	3.84	3.87	0.42	Postpartum	0.38	<0.01	0.45	0.40
	Tot	5.46	5.10		3.58	3.84							
β-carotene	CTR	0.56	0.53	0.55	0.26	0.31	0.27	0.05	Prepartum	0.29	<0.01	0.11	0.37
U/mL	TRT	0.55	0.46	0.53	0.29	0.28	0.29	0.05	Postpartum	0.98	<0.01	0.56	0.35
	Tot	0.56	0.50		0.28	0.30							
RFR	CTR	0.09	0.10	0.09	0.09	0.08	0.09	0.01	Prepartum	0.32	<0.01	0.87	0.20
-	TRT	0.09	0.09	0.09	0.09	0.09	0.09	0.01	Postpartum	0.71	0.23	0.99	0.27
	Tot	0.09	0.09		0.09	0.09							

[1]NEFA is nonesterified fatty acids; BHB is β-hydroxybutyrate; NAR is NEFA/albumin ratio = NEFA (mmol/L)/albumin (g/L); QUICKY is quantitative insulin sensitivity check index = 1/[Log$_2$ (Glucose (mmol/L) x 18.0182) + Log$_2$ Insulin (µU/ml)]; RQUICKY is revised quantitative insulin sensitivity check index = 1/[Log$_2$ (Glucose (mmol/L) x 18.0182) + Log$_2$ Insulin (µU/mL) + Log$_2$ NEFA (mmol/L)]; RQUICKY$_{BHB}$ is revised quantitative insulin sensitivity check index including BHB contribution = 1/[Log$_2$ (Glucose (mmol/L) x 18.0182) + Log$_2$ Insulin (µU/mL) + Log$_2$ NEFA (mmol/L) + Log$_2$ BHB (mmol/L)]; GGT is gamma glutamyl transferase; ALP is alkaline phosphatase; AGR is albumin to globulin ratio = Albumin (g/L)/ Globulin (g/L); MPR is myeloperoxidase to paraoxonase ratio = Myeloperoxidase (U/L) / Paraoxonase (U/mL); LFI is liver functionality index = [(Cholesterol RC + Albumin RC) – Bilirubin RC], where Cholesterol RC = {[0.5*Cholesterol 3 DIM+0.5*(Cholesterol 28 DIM – Cholesterol 3 DIM)]-2.57}/0.43; Albumin RC = {[0.5*Albumin 3 DIM+0.5*(Albumin 28 DIM – Albumin 3 DIM)]-17.71}/1.08; Bilirubin RC = {[0.67*Bilirubin 3 DIM+0.33*(Bilirubin 28 DIM – Bilirubin 3 DIM)]-4.01}/1.21; ROMt is total reactive oxygen species; GPx is glutathione peroxidase; FRAP is ferric ion reducing antioxidant power; RFR is ROMt to FRAP ratio = ROMt (mg H_2O_2/100mL) / FRAP (µmol/L)

[2]TRT is cow receiving 19 g/d of *Saccharomyces cerevisiae* fermentation product between -76 and 60 days from calving; CTR is cow receiving a control diet between -76 and 60 days from calving;

[3]Cows that had an average weekly SCC lower than 100 K/mL milk for primiparous and 200 K/mL milk for multiparous between -83 and -76 days from calving were classified as L; whereas cows that were above those thresholds were classified as H (CTR-L = 23 cows, CTR-H = 7 cows, TRT-L = 23 cows, TRT-H = 7 cows).

[4]Standard error = largest standard error for the fixed effects.

Table S4. Complete blood count in dairy cows having a low (L) or high (H) SCC between -83 and -76 days from calving and receiving 19 g/d of *Saccharomyces cerevisiae* fermentation product (TRT) or a control diet (CTR) from -76 thru 60 days from calving.

Item	TREAT [2]	Prepartum			Postpartum			SEM [4]	Period	P-VALUE			
		SCC_LV [3]		Tot	SCC_LV		Tot						
Unit [1]		L	H		L	H				TREAT	TIME	SCC_LV	TREAT X SCC_LV
MCV	CTR	52.13	51.43	51.97	53.02	52.07	52.80	1.27	Prepartum	0.85	<0.01	0.55	0.15
fL	TRT	50.78	52.47	51.17	52.43	53.40	52.66	1.27	Postpartum	0.72	0.01	0.99	0.35
	Tot	51.46	51.95		52.72	52.74							
MCH	CTR	33.22	33.38	17.28	32.60	32.78	17.14	0.44	Prepartum	0.96	0.27	0.59	0.33
Pg	TRT	33.44	33.31	17.09	32.38	33.00	17.10	0.44	Postpartum	0.52	0.01	0.67	0.15
	Tot	17.15	17.33		17.09	17.22							
MCHC	CTR	17.32	17.17	33.26	17.21	16.91	32.64	0.41	Prepartum	0.78	<0.01	0.96	0.59
g/dL	TRT	16.98	17.48	33.41	16.97	17.53	32.53	0.41	Postpartum	0.99	0.73	0.27	0.54
	Tot	33.33	33.34		32.49	32.89							
Platelets	CTR	270.2	324.1	282.8	347.2	384.7	356.0	30.3	Prepartum	0.33	<0.01	0.52	0.12
K/µL	TRT	332.1	309.5	326.9	397.7	403.6	399.1	30.3	Postpartum	0.16	<0.01	0.38	0.52
	Tot	301.2	316.8		372.5	394.1							
WBC	CTR	7.92	7.21	7.75	7.10	6.09	6.87	0.61	Prepartum	0.28	0.01	0.20	0.35
K/µL	TRT	7.97	7.85	7.94	6.84	7.09	6.90	0.61	Postpartum	0.46	0.64	0.45	0.21
	Tot	7.94	7.53		6.97	6.59							
Monocytes	CTR	11.67	11.77	11.69	16.61	14.58	16.14	1.37	Prepartum	0.46	0.02	0.61	0.73
%WBC	TRT	11.06	11.54	11.17	16.38	15.17	16.10	1.37	Postpartum	0.87	<0.01	0.15	0.71
	Tot	11.36	11.65		16.49	14.88							
Eosinophils	CTR	0.44	0.32	0.41	0.12	0.13	0.12	0.09	Prepartum	0.72	<0.01	0.29	0.45
K/µL	TRT	0.37	0.34	0.36	0.12	0.09	0.11	0.09	Postpartum	0.41	0.27	0.65	0.47
	Tot	0.40	0.33		0.12	0.11							
Eosinophils	CTR	5.59	4.58	5.36	1.74	2.01	1.80	0.98	Prepartum	0.45	<0.01	0.37	0.70
%WBC	TRT	4.68	4.28	4.59	1.79	1.37	1.69	0.98	Postpartum	0.41	0.19	0.84	0.34
	Tot	5.14	4.43		1.76	1.69							
Neutrophils	CTR	3.58	3.67	3.60	3.23	2.75	3.12	0.46	Prepartum	0.32	<0.01	0.38	0.69
K/µL	TRT	3.69	3.91	3.74	2.95	3.39	3.05	0.46	Postpartum	0.63	0.08	0.95	0.22
	Tot	3.64	3.79		3.09	3.07							

[1]MCV is mean cell volume, MCH is mean cell hemoglobin, MCHC is mean cell hemoglobin concentration, WBC is white blood cells;
[2]TRT is cow receiving 19 g/d of saccharomyces cerevisiae fermentation product between -76 and 60 days from calving; CTR is cow receiving a control diet between -76 days and 60 days from calving.

[3]L is cow having an average SCC level lower than safety thresholds (100 K/mL milk for primiparous and 200 K/mL milk for multiparous) between -83 and -76 days from calving; H is cow having an average SCC level higher than safety thresholds (100 K/mL milk for primiparous and 200 K/mL milk for multiparous) between -83 and -76 days from calving (CTR L = 23 cows, CTR H = 7 cows, TRT L = 23 cows, TRT H = 7 cows).

[4]Standard error = largest standard error for the fixed effects.
